# Nobiletin enhances the development and quality of bovine embryos in vitro during two key periods of embryonic genome activation

**DOI:** 10.1038/s41598-021-91158-7

**Published:** 2021-06-03

**Authors:** Karina Cañón-Beltrán, Yulia N. Cajas, Serafín Peréz-Cerezales, Claudia L. V. Leal, Ekaitz Agirregoitia, Alfonso Gutierrez-Adán, Encina M. González, Dimitrios Rizos

**Affiliations:** 1 Department of Animal Reproduction, National Institute for Agriculture and Food Research and Technology (INIA), 28040 Madrid, Spain; 2 Departamento de Ciencias Biológicas, Universidad Técnica Particular de Loja, Loja, 110107 Ecuador; 3 Department of Veterinary Medicine, Faculty of Animal Science and Food Engineering, University of São Paulo, Pirassununga, 13635-900 Brazil; 4 Department of Physiology, Faculty of Medicine and Nursing, Universidad del País Vasco/Euskal Herriko Unibertsitatea (UPV/EHU), 48940 Bizkaia, Spain; 5 Department of Anatomy and Embryology, Veterinary Faculty, Complutense University of Madrid (UCM), 28040 Madrid, Spain

**Keywords:** Biotechnology, Developmental biology, Molecular biology

## Abstract

In vitro culture can alter the development and quality of bovine embryos. Therefore, we aimed to evaluate whether nobiletin supplementation during EGA improves embryonic development and blastocyst quality and if it affects PI3K/AKT signaling pathway. In vitro zygotes were cultured in SOF + 5% FCS (Control) or supplemented with 5, 10 or 25 µM nobiletin (Nob5, Nob10, Nob25) or with 0.03% dimethyl-sulfoxide (C_DMSO_) during minor (2 to 8-cell stage; MN_EGA_) or major (8 to 16-cell stage; MJ_EGA_) EGA phase. Blastocyst yield on Day 8 was higher in Nob5 (42.7 ± 1.0%) and Nob10 (44.4 ± 1.3%) for MN_EGA_ phase and in Nob10 (61.0 ± 0.8%) for MJ_EGA_ phase compared to other groups. Mitochondrial activity was higher and lipid content was reduced in blastocysts produced with nobiletin, irrespective of EGA phase. The mRNA abundance of *CDK2, H3-3B, H3-3A, GPX1, NFE2L2* and *PPARα* transcripts was increased in 8-cells, 16-cells and blastocysts from nobiletin groups. Immunofluorescence analysis revealed immunoreactive proteins for p-AKT forms (Thr308 and Ser473) in bovine blastocysts produced with nobiletin. In conclusion, nobiletin supplementation during EGA has a positive effect on preimplantation bovine embryonic development in vitro and corroborates on the quality improvement of the produced blastocysts which could be modulated by the activation of AKT signaling pathway.

## Introduction

In vitro culture (IVC) of bovine embryos is one of the most important processes in the development of assisted reproductive techniques due to the fact that postfertilization culture conditions can dramatically alter the quality of the resulting blastocysts^1,2^. In vitro, gametes and embryos are exposed to spatial and temporal unnatural conditions, whose scope is not completely known^3^. Although many improvements have been made, in vitro culture systems are still not as efficient as in vivo embryo production^4^. In cattle, the proportion of embryos reaching the blastocyst stage is around 30–40%^5^ and are often compromised in quality and competence manifested by a darker morphology^1^ or altered gene expression patterns^6^ when compared to their *in vivo* counterparts. The factors that most influence the quality of the embryos are the conditions after fertilization; which include physicochemical (temperature, osmolality, and pH), oxidative (antioxidant balance), and energetic (production, utilization, and storage) stresses^6^.

Under in vitro conditions, the dynamics of embryo development and the kinetics of cleavage are related to the subsequent developmental stages: the faster-cleaved embryos have a higher chance to develop to the blastocysts stage^7^. Therefore, the morphological and metabolic changes that occur during the first 4 days of preimplantation development of the bovine embryo are the most important; besides, during this same period the embryonic genome activation (EGA) occurs^2,8^. At the start of early embryogenesis, all mRNAs and proteins controlling development are of maternal origin, and as development progresses, these reserves gradually degrade while embryonic transcripts are synthesized; this process is called maternal-to-embryonic transition and involves EGA^9^. The EGA occurs in distinct waves, which are species-specific. Bovine preimplantation embryo development is characterized by two distinct phases: (i) minor EGA (MN_EGA_) (2-cell to 8-cell stages) where zygotes and early embryos are transcriptionally and translationally active; (ii) major EGA (MJ_EGA_) (8-cell to 16-cells stages) which includes a gradual degradation of mRNA molecules of maternal origin, together with a change in the protein synthesis, and these events are key factors for successful embryonic development and differentiation^2,8^. EGA is a prerequisite for correct compaction, that leads to an increase in intercellular adhesion mediated by adherent junctions and embryonic polarization^10^, as well as the formation of the blastocyst, with its trophectoderm (TE), and the inner cell mass (ICM)^11^.

In recent years, the role of different signaling pathways in preimplantation development has been analyzed, suggesting the existence of a complex network of signals that control and are responsible for cell division, differentiation, cytoskeleton rearrangements, cell proliferation and apoptosis^11,12^. One of the most important signal transduction pathways that regulate cell survival is PI3K/AKT. PI3K/AKT pathway consists of several molecules, including kinases, phosphatases, and transcription factors that are fundamental in processes such as migration, metabolism, and cell cycle progression^13,14^. During embryonic development, PI3K/AKT regulates cell survival and its inhibition can cause a significant delay in blastocyst hatching^12^. In this context, the quality of the embryos produced in vitro depends not only on the proper functioning of the signaling pathways but also on the post-fertilization culture environment.

To improve the blastocysts rates and quality, several studies have probed the addition of different types of natural antioxidants to the IVC medium, such as vitamin C^15^ or crocetin^16^. These compounds improved embryonic quality in terms of increase in blastocysts rates and embryo cell number, as well as reduction in reactive oxygen species (ROS) levels and apoptotic cells in embryos. In recent years, nobiletin a class of polymethoxylated flavone identified from the citrus peel (chemically known as 5,6,7,8,3′,4′ hexamethoxyflavone), has drawn increasing attention since it is easily absorbed across the cytoplasmic membranes due to its structure and lipophilic nature^17^. In addition, it has been reported that nobiletin has a broad spectrum of biological activities, that include antioxidative functions and cell cycle regulation^17^. We observed that supplementation of *in vitro* maturation (IVM) medium with nobiletin counteracts the effects of the increase in ROS production during IVM, improves oocyte nuclear and cytoplasmic maturation, and subsequent embryo development and quality in bovine^18^. Other studies using cultured cell lines have demonstrated that nobiletin can modulate signaling cascades, including PI3K/AKT signaling pathway^17,19^. Nevertheless, the mechanism of specific action by which nobiletin modulates this signaling pathway is not fully understood, and, to our knowledge, there is no evidence of any developmental effect of nobiletin supplementation during post-fertilization embryo culture in vitro. Thus, in this study, we aimed to evaluate whether supplementation of nobiletin to the in vitro culture medium during the two EGA phases improves embryonic development and blastocyst quality and if its action is related to the PI3K/AKT signaling pathway. The parameters evaluated in blastocysts were, (i) lipid accumulation, (ii) mitochondrial activity, (iii) quantitative changes of key genes related to quality and development, (iv) immunolocalization of phosphorylated-AKT (p-AKT) and (v) level by western blot analysis for AKT and p-AKT (Thr308/Ser473).

## Results

### Nobiletin during MN_EGA_ or MJ_EGA_ enhances early embryo development in vitro

**Figure 1:**
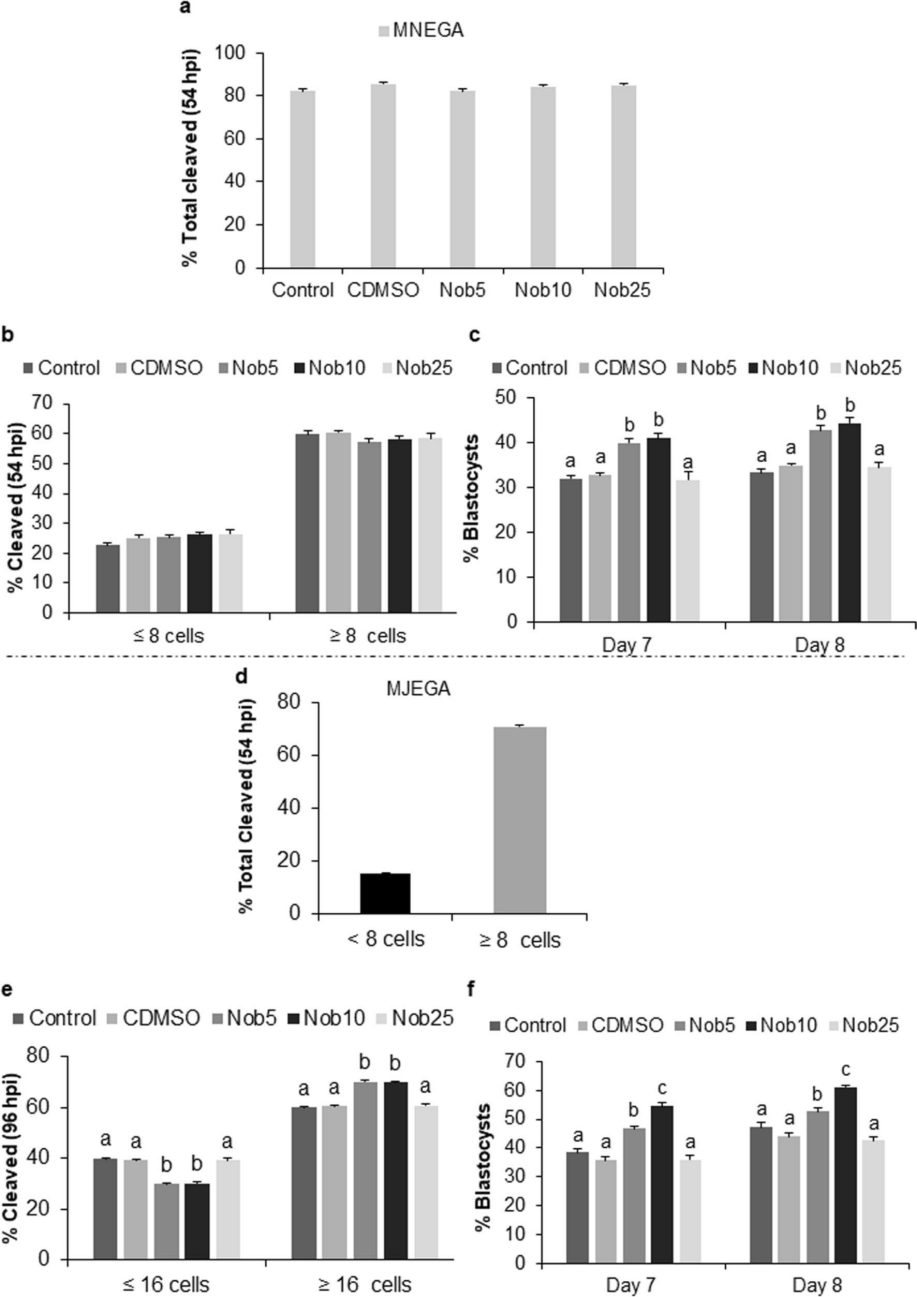
Nobiletin effect in embryonic development. Developmental rates of in vitro produced bovine embryos cultured during 21–54 h post-insemination (hpi) (MN_EGA_: **a**–**c**) or during 54–96 hpi (MJ_EGA_: **d**–**f**) with or without nobiletin. (**a**,**d**) Total cleavage rate at 54 hpi; (**b,e**) embryos ≥ or ≤ 8-cell stage at 54 hpi and ≥ or ≤ 16-cell stage at 96 hpi; (**c**,**f**) blastocyst rate on Days 7–8 pi (in vitro fertilization = Day 0), from embryos cultured in SOF + 5% FCS (Control), supplemented or not with 5 (Nob5), 10 (Nob10) or 25 µM (Nob25) nobiletin or with 0.03% dimethyl sulfoxide (C_DMSO_) during MN_EGA_ or MJ_EGA_ respectively. Results are expressed as mean ± s.e.m. Significant differences (*P* < 0.001) are indicated with different letters.

For all experimental groups, only embryos that reached the 8-cell stage at 54 h post-insemination (hpi) were selected for the study. As shown in Fig. 1a for MN_EGA_ phase, no differences were observed in cleavage rate at 54 hpi, which ranged from 82.3 ± 1.0 to 85.5 ± 0.5%. At 54 hpi, no differences were observed either in the proportion of embryos that reached the 8-cell stage, which ranged from 57.1 ± 1.4 to 60.4 ± 0.7%. Consequently, a similar proportion of embryos with a delayed development (< 8 cells), which ranged from 22.6 ± 0.9 to 26.6 ± 1.2%, was observed (Fig. 1b). Blastocyst yield at Day 7 and 8 (Fig. 1c) was significantly higher (*P *< 0.001) for Nob5 (39.7 ± 0.8 and 42.7 ± 1.0%, respectively) and Nob10 (41.0 ± 1.0 and 44.4 ± 1.3%), compared to Control (32.7 ± 0.7 and 34.6 ± 0.7%); C_DMSO_ (32.8 ± 0.5 and 34.9 ± 0.4%) and Nob25 (31.8 ± 1.7 and 34.6 ± 1.2%) (Supplementary Table S1).

During MJ_EGA_, cleavage rate at 54 hpi was 86.6 ± 0.2% and the proportion of embryos that reached the 8-cell stage was 71.1 ± 0.4% while the proportion of embryos with a delayed development (< 8 cells) was 15.5 ± 0.3% (Fig. 1d). At 96 hpi a significantly (*P *< 0.001) higher proportion of embryos reached the 16-cell stage in Nob5 and Nob10 groups (70.1 ± 0.5% and 69.9 ± 0.4%, respectively) compared to Control (60.0 ± 0.4%), C_DMSO_ (60.7 ± 0.4%) and Nob25 (60.8 ± 0.8%) groups (Fig. 1e). As a consequence, a significantly lower proportion of embryos with a delayed development (<16 cells) was observed in Nob5 and Nob10 compared to the other groups (Nob5: 29.9 ± 0.5% and Nob10: 30.1 ± 0.4% vs Control: 40.0 ± 0.4%, C_DMSO_: 39.3 ± 0.4% and Nob25: 39.2 ± 0.8%, *P* < 0.001). On Day 7 and 8, blastocyst yield was significantly higher (*P* < 0.001) for Nob10 (54.5 ± 1.1% and 61.0 ± 0.8%, respectively) compared to Control (38.4 ± 1.1% and 47.3 ± 1.4%), C_DMSO_ (35.8 ± 1.0% and 44.0 ± 1.1%), Nob5 (46.6 ± 0.8% and 52.5 ± 1.5%) and Nob25 (35.9 ± 1.5%–42.5 ± 1.3%) groups, while Nob5 was higher (*P *< 0.001) compared to Nob25 and both control groups (Fig. 1f) (Supplementary Table S2).

### Nobiletin during MN_EGA_ or MJ_EGA_ increases the quality of in vitro produced blastocysts

Only the experimental groups that showed better blastocyst yield in the previous experiment (Nob5 and Nob10 during MN_EGA_ or MJ_EGA_) were used for embryo quality evaluation in comparison with both control groups (Control and C_DMSO_).

**Figure 2:**
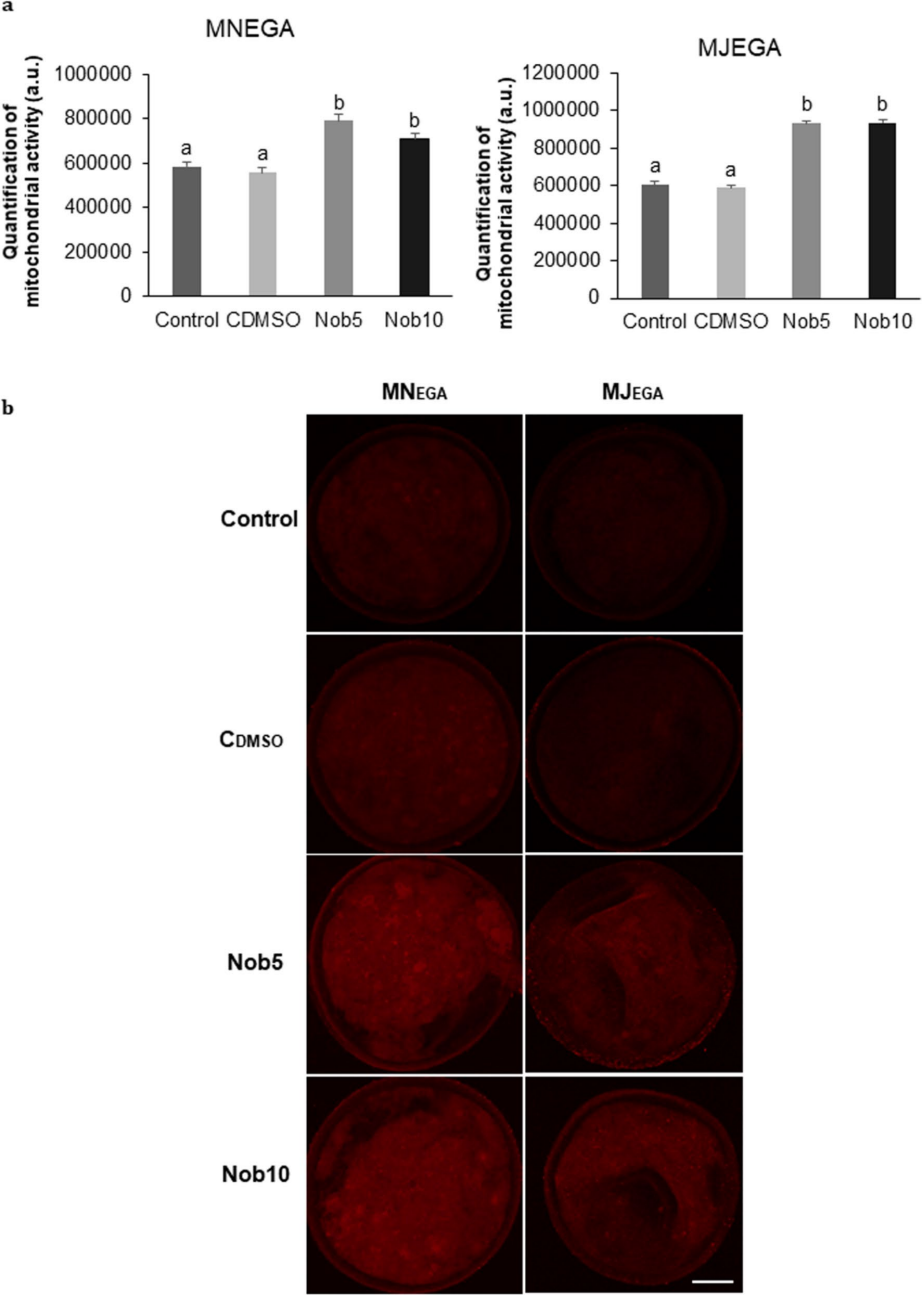
Nobiletin effect in blastocysts mitochondrial activity. (**a**) Quantification of mitochondrial fluorescence intensity in arbitrary units (a.u) in Day 7 blastocysts cultured in SOF + 5% FCS (Control), supplemented or not with 5 (Nob5) or 10 µM (Nob10) nobiletin or with 0.03% dimethyl sulfoxide (C_DMSO_) during 21–54 hpi (MN_EGA_: presumptive zygote to 8-cell stage) or during 54–96 hpi (MJ_EGA_: 8- to 16-cell stage). Data are the mean ± s.e.m. Significant differences (*P* < 0.001) are indicated with different letters. (**b**) Representative fluorescence images of mitochondrial activity in Day 7 blastocysts from all experimental groups (Control, Nob5 Nob10, C_DMSO_) in both phases (MN_EGA_ or MJ_EGA_). Images were captured on 63× objective. Scale bar 50 µm.

The mitochondrial activity was higher (*P *< 0.001) in blastocysts from Nob5 and Nob10 groups, from either MN_EGA_ or MJ_EGA_ phase, compared with both control groups (Fig. 2).

**Figure 3:**
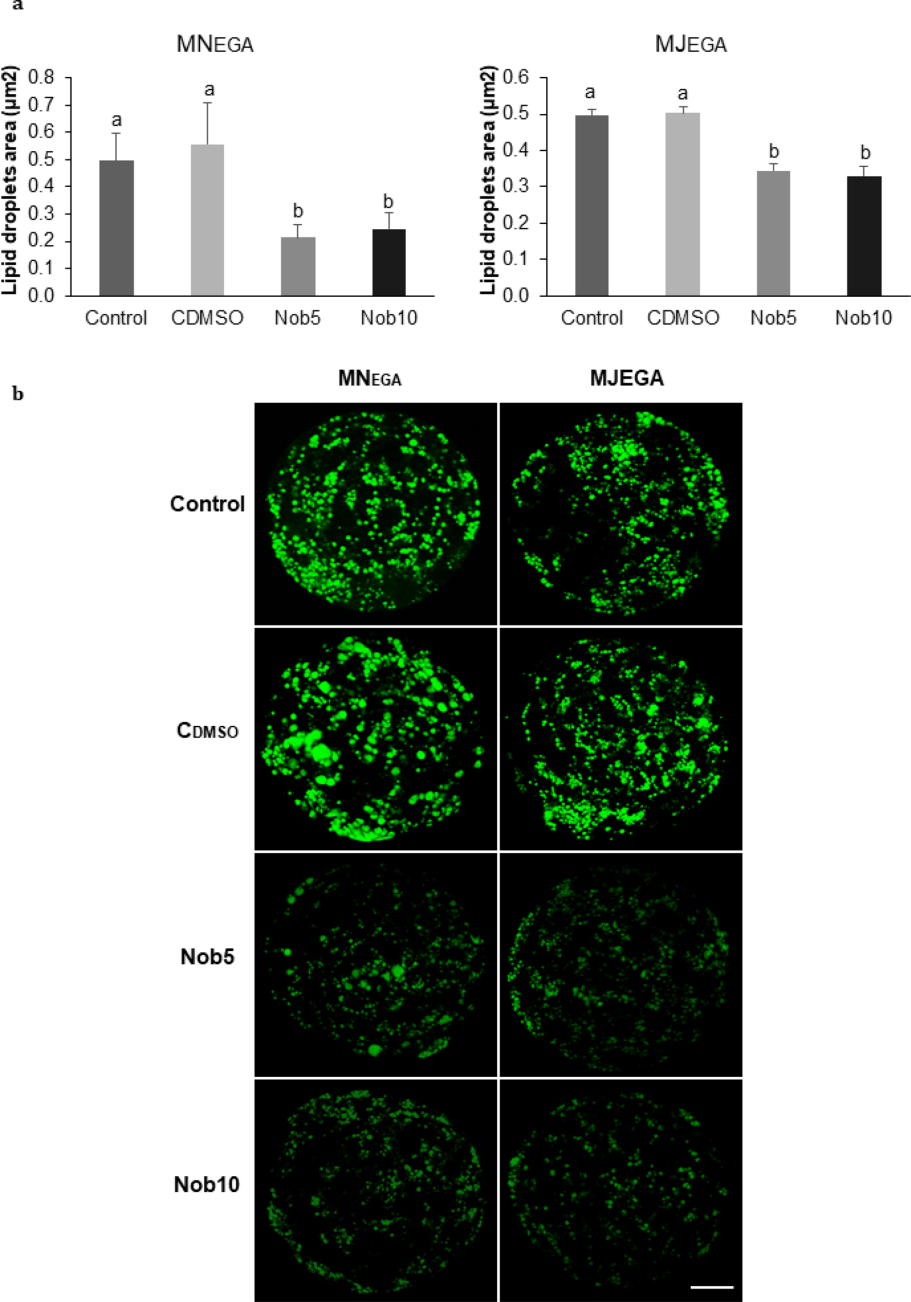
Nobiletin effect in blastocysts lipid content. (**a**) Quantification of the total area of lipid droplets (µm^2^) in Day 7 blastocysts cultured in SOF + 5% FCS (Control), supplemented or not with 5 (Nob5) or 10 µM (Nob10) nobiletin or with 0.03% dimethyl sulfoxide (C_DMSO_) during 21–54 hpi (MN_EGA_: presumptive zygote to 8-cell stage) or during 54–96 hpi (MJ_EGA_: 8- to 16-cell stage). Data are the mean ± s.e.m. Significant differences (*P* < 0.001) are indicated with different letters. (**b**) Representative fluorescence images of lipid droplets in Day 7 blastocysts from all experimental groups (Control, Nob5 Nob10, C_DMSO_) in both phases (MN_EGA_ or MJ_EGA_). Images were captured on 63× objective. Scale bar 50 µm.

When analyzing the lipid content, we observed that the total area of lipid droplets in blastocysts resulting from treatments during MN_EGA_ or MJ_EGA_ was significantly reduced (*P *< 0.001) in Nob5 and Nob10 groups compared with the control groups (Fig. 3).

The total number of cells was greater (*P *< 0.001) in blastocysts from MN_EGA_ phase produces with 5 µM of nobiletin (137.3 ± 0.6) compared to all other groups (Control: 105.7 ± 0.7; C_DMSO_: 106.4 ± 0.8; Nob10: 126.7 ± 0.8), while blastocysts from Nob10 group had more cells (*P *< 0.001) compared to control groups, but less (*P *< 0.001) when compared to Nob5. However, during MJ_EGA_ phase the total number of cells was higher in blastocysts from Nob5 and Nob10 groups (133.2 ± 0.9 and 134.2 ± 0.7, respectively) compared to control groups (Control: 104.9 ± 0.7 and C_DMSO_: 104.6 ± 0.6) (*P *< 0.001) (Supplementary Table S3).

#### Gene expression in ≥ 8-cell embryos and blastocysts produced with nobiletin during MN_EGA_

**Figure 4:**
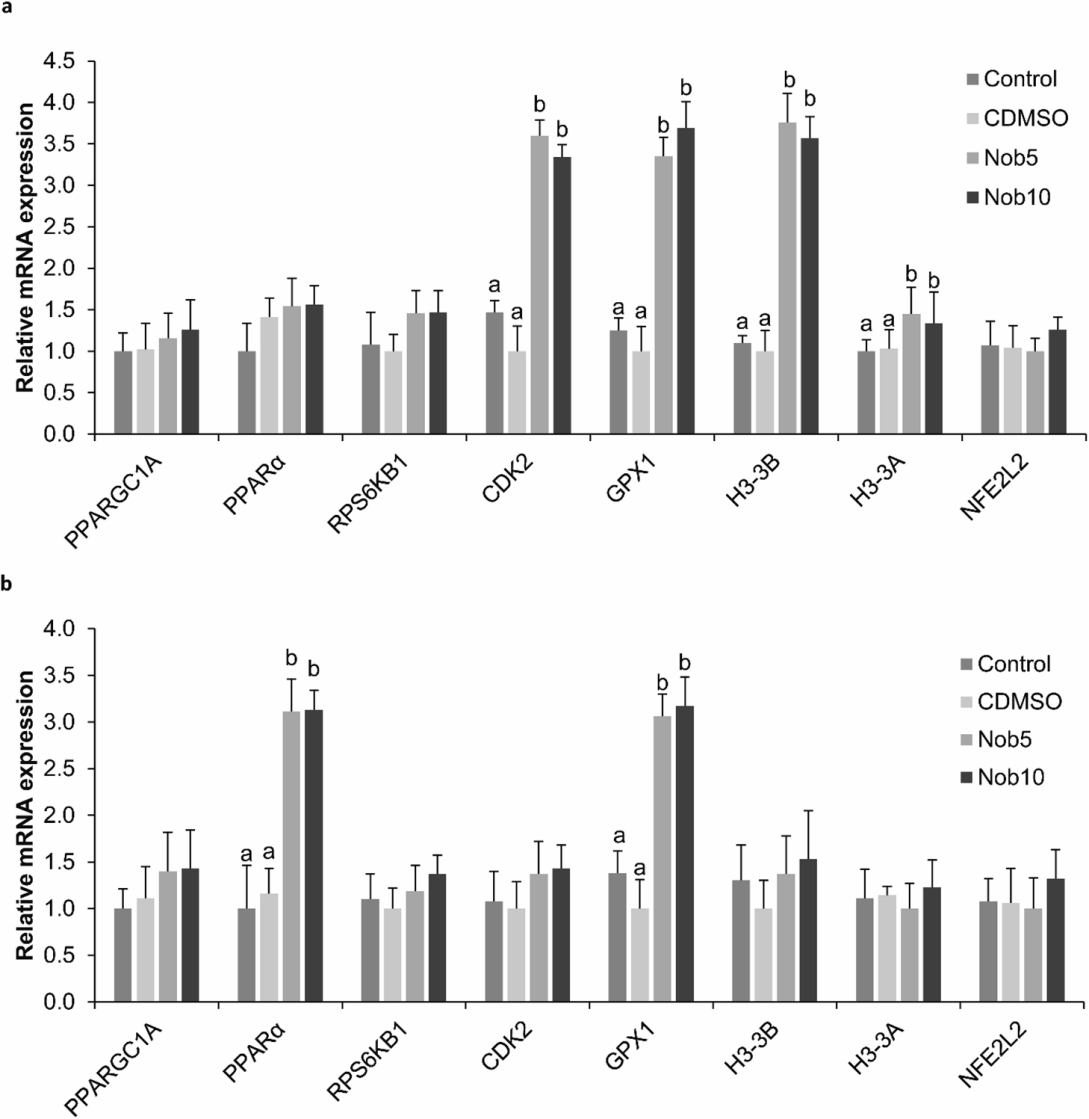
Relative mRNA transcript abundance of embryo development-related genes in in vitro produced embryos cultured during 21–54 h post insemination (MN_EGA_: presumptive zygote to 8-cell stage) with or without nobiletin. (**a**) Relative abundance in 8-cell stage embryos cultured in SOF + 5% FCS (Control), supplemented or not with 5 (Nob5) or 10 µM (Nob10) nobiletin or with 0.03% dimethyl sulfoxide (C_DMSO_) during MN_EGA_ phase. (**b**) Relative abundance in blastocysts from Control, Nob5, Nob10, and C_DMSO_ experimental groups from MN_EGA_ phase. The relative abundance of the transcripts was normalized to *H2AFZ* and *ACTB* as housekeeping genes. Data are the mean ± s.e.m. Different letters indicate significant difference (*P* < 0.05) between treatments.

The mRNA abundance of *CDK2, H3-3B, H3-3A,* and *GPX1* was significantly increased in 8-cell stage embryos from Nob5 and Nob10 groups compared to both controls (*P *< 0.05) (Fig. 4a). The expression of *PPARα* and *GPX1* was significantly higher in blastocysts from Nob5 and Nob10 groups when compared with both controls (*P *< 0.05) (Fig. 4b). No differences were observed for the *PPARGC1A, PPARα, RPS6KB1,* and *NFE2L2* transcripts in 8-cell stage embryos and *PPARGC1A, RPS6KB1, CDK2, H3-3B, H3F3A,* and *NFE2L2* in blastocysts.

#### Gene expression in ≥ 16-cell embryos and blastocysts produced with nobiletin during MJ_EGA_

**Figure 5:**
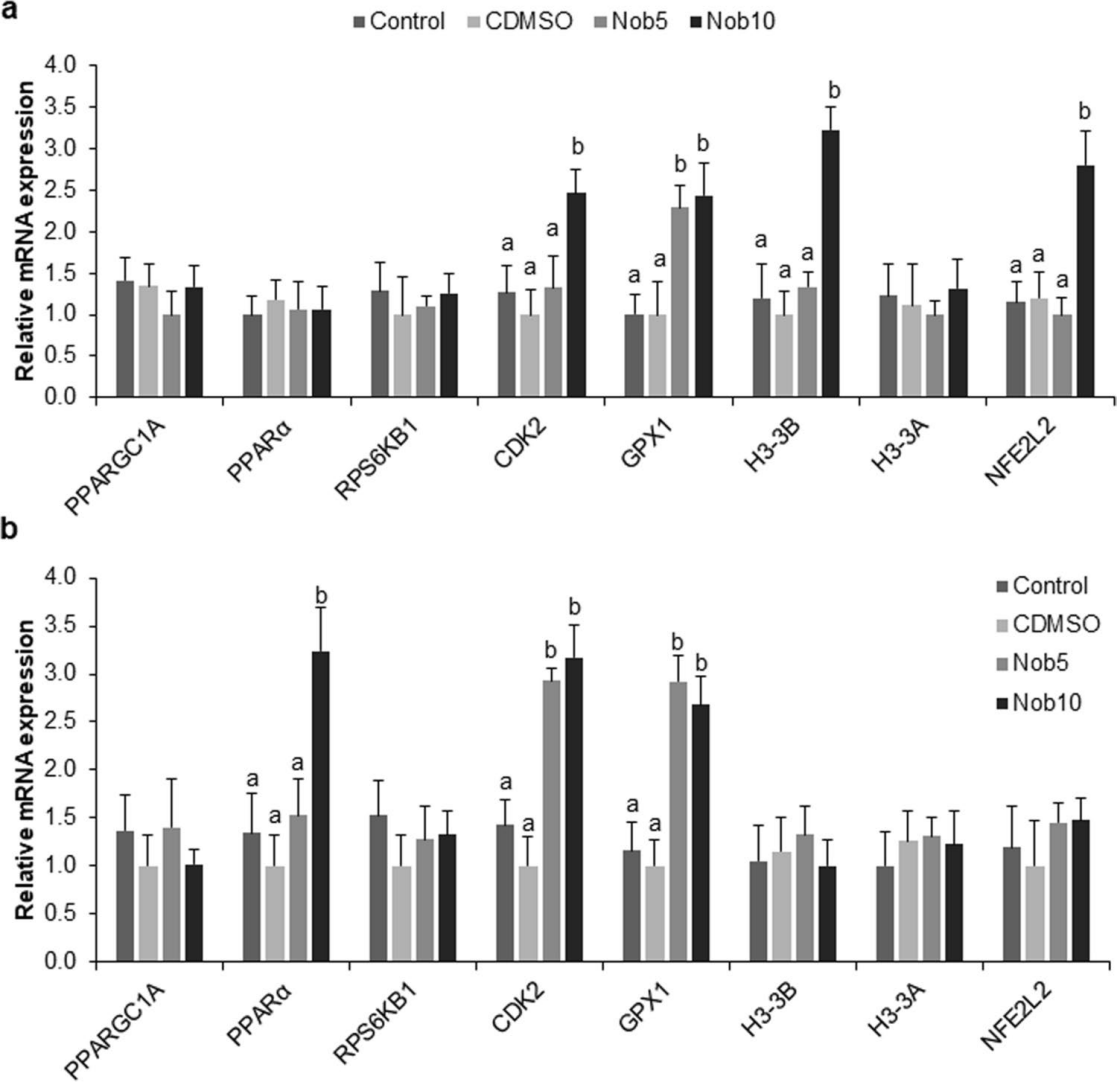
Relative mRNA transcript abundance of embryo development-related genes in in vitro produced embryos cultured during 54–96 hpi (MJ_EGA_: 8-to 16-cell stage) with or without nobiletin. (**a**) Relative abundance in16-cell stage embryos cultured in SOF + 5% FCS (Control), supplemented or not with 5 (Nob5) or 10 µM (Nob10) nobiletin or with 0.03% dimethyl sulfoxide (C_DMSO_) during MN_EGA_ phase. (**b**) Relative abundance in blastocysts from Control, Nob5, Nob10, and C_DMSO_ experimental groups from MJ_EGA_ phase. The relative abundance of the transcripts was normalized to *H2AFZ* and *ACTB* as housekeeping genes. Data are the mean ± s.e.m. Different letters indicate significant difference (*P* < 0.05) between treatments.

The expression level of *CDK2, H3-3B* and *NFE2L2* transcripts was significantly increased in 16-cell stage embryos from Nob10 group compared to Nob5 and both control groups. While the expression of *GPX1* gene was higher in Nob5 and Nob10 compared to control groups (*P *< 0.05) (Fig. 5a). In blastocysts the expression of *PPARα* was significantly higher in Nob10 group compared to all other groups (*P* < 0.05), while *CDK2* and *GPX1* were upregulated in both nobiletin groups compared with controls (*P* < 0.05) (Fig. 5b). No significant differences were observed for *PPARGC1A, PPARα, RPS6KB1* and *H3-3A* in 16-cell stage embryos, and for *PPARGC1A, RPS6KB1, H3-3B, H3-3A* and *NFE2L2* in blastocysts.

### Nobiletin during MN_EGA_ or MJ_EGA_ increases AKT phosphorylation in blastocysts produced in vitro

**Figure 6:**
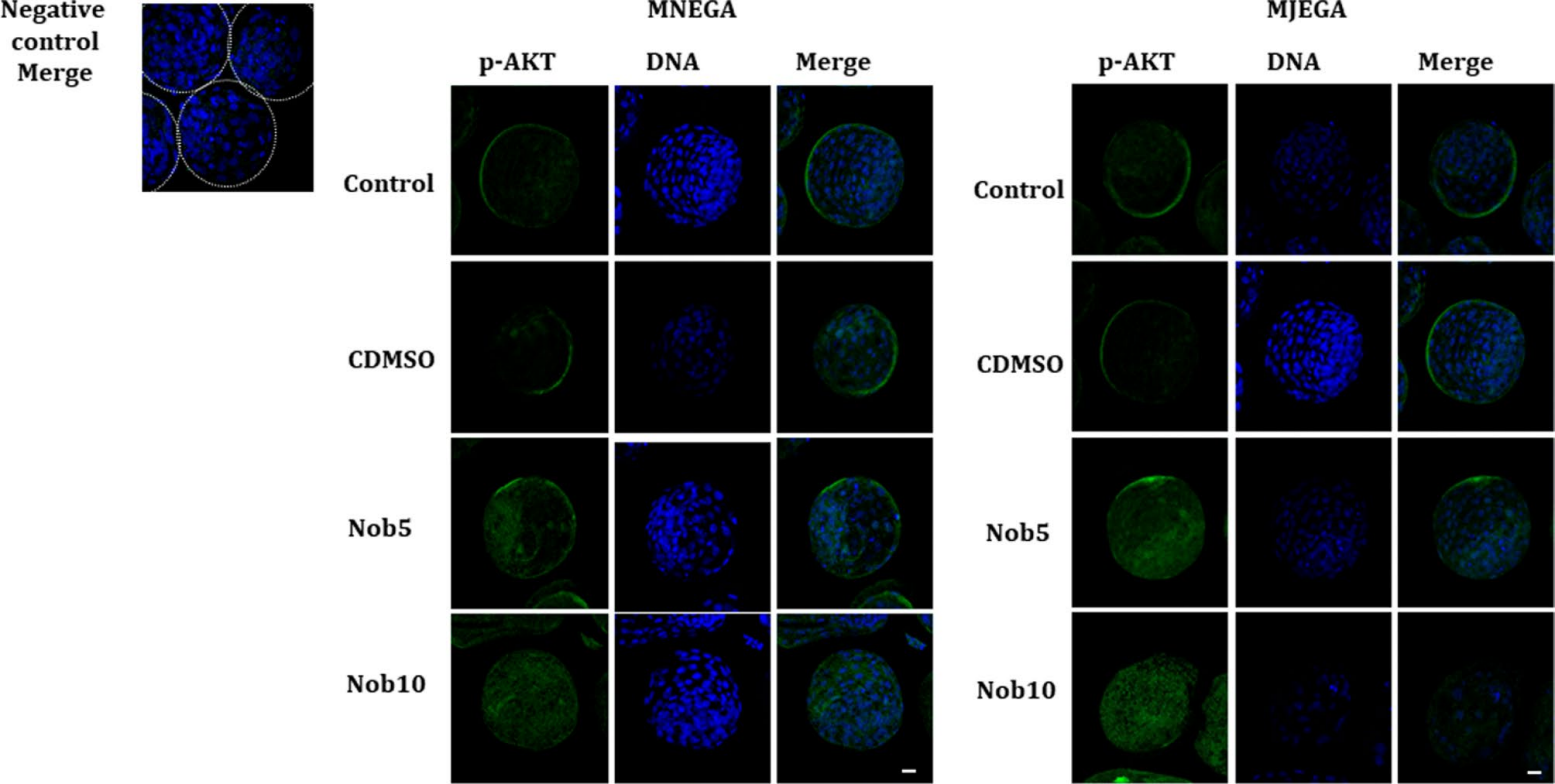
Nobiletin effect in the phosphorylated-AKT (p-AKT) in blastocysts. Representative images of immunofluorescence detection of p-AKT in in vitro produced bovine blastocysts cultured during 21–54 hpi (MN_EGA_: presumptive zygote to 8-cell stage) or during 54–96 hpi (MJ_EGA_: 8- to 16-cell stage) in SOF + 5% FCS (Control), supplemented or not with 5 (Nob5) or 10 µM (Nob10) nobiletin or with 0.03% dimethyl sulfoxide (C_DMSO_). Positive staining for p-AKT proteins shown in green. Cell nuclei were counterstained with Hoechst stain (blue). Images were captured on 63× objective. Scale bar 20 µm.

Immunofluorescence analysis revealed immunoreactive proteins for p-AKT in bovine blastocysts. In Day 7 blastocysts, AKT increased its phosphorylation levels when nobiletin was present in the culture medium (Nob5 and Nob10 groups) during MN_EGA_ or MJ_EGA_. While p-AKT levels were weaker in blastocysts produced from control groups during MJ_EGA_ phase (Fig. 6).

**Figure 7:**
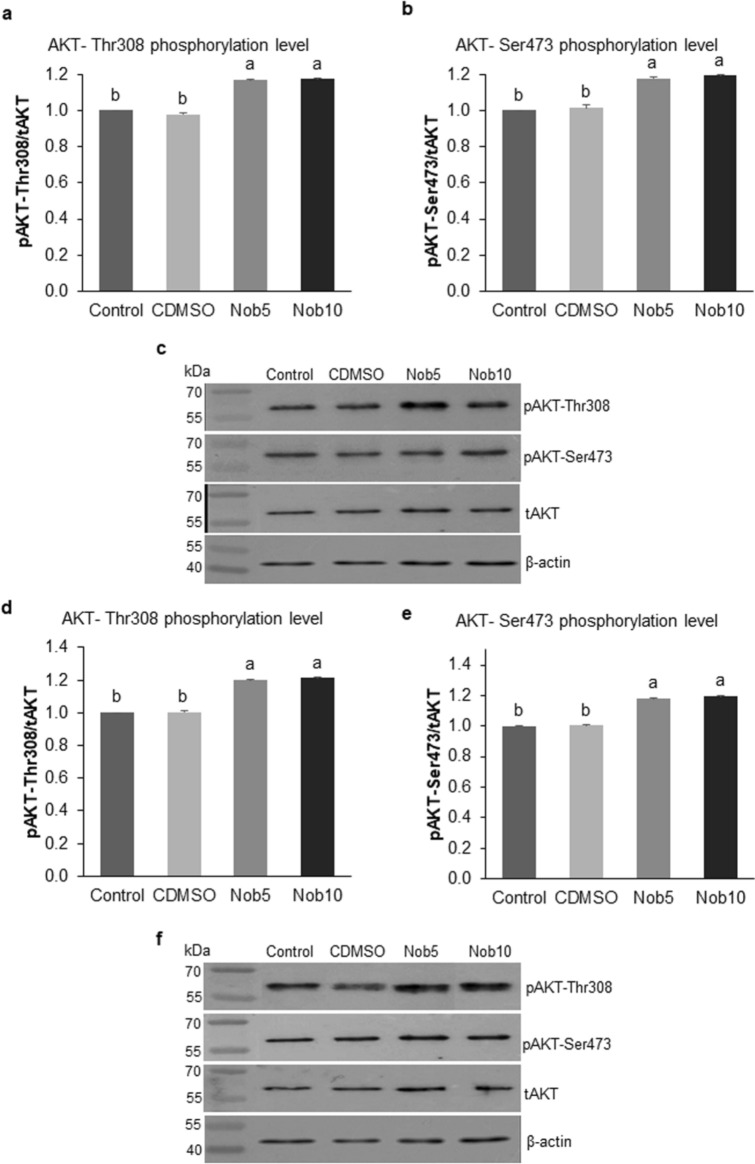
Nobiletin during MN_EGA_ or MJ_EGA_ increases AKT phosphorylation in blastocysts. (**a**,**b**) Quantification of phosphorylation levels of pAKT-Thr308/tAKT and pAKT-Ser473/tAKT in in vitro produced bovine Day 7 blastocysts cultured during 21–54 hpi (MN_EGA_: presumptive zygote to 8-cell stage) in SOF + 5% FCS (Control), supplemented or not with 5 µM (Nob5) or 10 µM (Nob10) nobiletin or with 0.03% dimethyl sulfoxide (C_DMSO_). (**c**) Western blot images showing the expression of pAKT-Thr308, pAKT-Ser473, (t)AKT and β-actin in Day 7 blastocysts from MN_EGA_ phase. (**d**,**e**) Quantification of phosphorylation levels of pAKT-Thr308/tAKT and pAKT-Ser473/tAKT in in vitro produced bovine Day 7 blastocysts cultured during 54–96 hpi (MJ_EGA_: 8- to 16-cell stage) in Control, Nob5, Nob10, and C_DMSO_. (**f**) Western blot images showing the expression of pAKT-Thr308, pAKT-Ser473, (t)AKT and β-actin in Day 7 blastocysts from MJ_EGA_ phase. Data were normalized relative to the abundance of β-actin and p-AKT phosphorylation levels. Samples derive from the same experiment and gels were processed in parallel. Cropped western blot membrane images are shown here, while full-length blots are presented in Supplementary Figure S1. Data are the mean ± s.e.m. Different letters indicate significant difference (*P* < 0.05) between treatments.

Similarly, the western blot analysis showed that both p-AKT-Thr308 and p-AKT-Ser473 phosphorylation levels were significantly higher in blastocysts produced with nobiletin supplementation (Nob5 and Nob10) during MN_EGA_ phase when compared with control groups (*P* < 0.05) (Fig. 7a–c). A similar pattern was observed in response to nobiletin treatment during MJ_EGA_, as p-AKT-Thr308 and p-AKT-Ser473 phosphorylation levels were significantly higher in blastocysts produced with Nob5 and Nob10 compared with control groups (*P* < 0.05) (Fig. 7d–f).

## Discussion

Under in vivo conditions, cells have antioxidants levels in equilibrium and possess physiological mechanisms to hinder excessive free radical formation^20^. During in vitro culture this mechanism suffers disturbances, in which the redox balance is altered with an increase in the production of free radicals and, as a consequence, a decrease in embryo development^6^. Several studies, aiming to identify the most effective antioxidants to reduce the alteration of the redox balance and ROS levels during the in vitro production of embryos, have shown that the addition of quercetin, resveratrol, vitamin C or carnitine to the culture media have beneficial effects on early embryonic development^15,21^. To our knowledge, the present study is the first that investigates the antioxidant effects of nobiletin supplementation in the culture medium during the two main phases of EGA (MN_EGA_: minor activation from 2- to 8-cell stage and MJ_EGA_: major activation from 8- to 16-cell stage)^2,8^ in bovine embryo developmental competence in vitro and quality of the produced blastocysts, as well as its possible interaction with the AKT signaling pathway.

Irrespective of concentration, addition of nobiletin to culture media during MN_EGA_ phase (21–54 hpi) did not affect cleavage rates at 54 hpi as well as the percentage of embryos reach the 8-cell stage but increased blastocyst production, whereas nobiletin supplementation in culture media during MJ_EGA_ phase (54–96 hpi) significantly increased the percentage of embryos that reach the16-cell stage and blastocyst production. Several studies have shown that during EGA the bovine embryo actively synthesize transcription factors and this process directly links to chromatin changes, protein allocation, nuclear reorganization and cell proliferation^8,22^. Since in our results developmental kinetics were stimulated with more 16-cell embryos by nobiletin during MJ_EGA_, we could hypothesize that nobiletin activates early embryonic genes important for the proper genomic function of the embryo during major EGA. Although with our experimental design we cannot link this effect specifically with either of the two activation phases of the embryonic genome.

The evidence that nobiletin supplementation improves blastocyst production is in line with other studies showing increased embryo development in vitro when culture medium was supplemented with biological antioxidants similar to nobiletin^16,23,24^. Another effect of nobiletin was to induce a significant increase in mitochondrial activity and a lower content of lipid droplets in blastocysts from both EGA phases analyzed. Mitochondria play a central role in the generation of adenosine triphosphate (ATP), so, they are considered as energy control units necessary for cell division, pluripotency and differentiation^25^. Cagnone and Sirard^6^, reported that in vivo, during the early cleavages, mitochondria and intracellular metabolism are quiescent. Nevertheless, in vitro, this metabolic quiescence is altered due to the presence of nutrients in excessive amounts that overstimulate mitochondria and alter their efficiency to respond to oxidative phosphorylation^6^. Mitochondria also sense changes in redox potential and force embryos to adapt versus the decreased production of ATP by oxidative phosphorylation during the transition from morula to blastocyst^6,25^. Besides, some studies reported that changes in mitochondrial activity may affect the development of energetic metabolism in the embryo, in terms of availability of glucose, lipids, amino acids and DNA methylation^6,26^. Although the nobiletin action mechanism in mitochondria has not been fully elucidated, in a previous study we observed that increased oocyte mitochondrial activity was related to the cytoprotective effects of nobiletin and its intrinsic ROS-scavenging property^18^. Nevertheless, the effect in the blastocyst could be explained based on the fact that nobiletin is a hydrophobic compound, which easily penetrates through cell membranes directly affecting mitochondrial bioenergetics. Nobiletin can modify intramitochondrial proteins (e.g. acetylated proteins localized within the mitochondria in the brain of rats)^27^ or alter the mitochondrial membrane potential by changing the activities of mitochondrial enzymes, like succinate dehydrogenase and cytochrome c oxidase as it has been demonstrated in human blood lymphocytes^28^. However, to verify if this mechanism occurs in bovine blastocysts, further investigation is necessary.

Lipid content is a crucial factor for early embryo development in vitro in bovine since energy metabolism is abnormal under such conditions, resulting in an excessive accumulation of lipids associated with reduced embryonic quality^29^. Lipids are stored in intracellular droplets and are metabolized via β-oxidation in the mitochondrial matrix. A large amount of lipid droplets increases the production of ATP necessary for the formation of blastocysts but this can affect its quality; thus, a lower number of lipid droplets in blastocysts is considered as a criterion of good quality embryos^16^. Our results showed for both EGA phases, nobiletin supplementation in culture medium reduced the amount of lipids in blastocysts. Furthermore, we analyze the expression of peroxisome proliferator-activated receptor alpha transcript (*PPARα),* belonging to one of the 3 key nuclear receptors in the modulation of transcription for lipid metabolism-related genes^30^. *PPARα* was previously detected in cattle embryos and has been associated with embryo quality^31^. In our study, *PPARα* was significantly upregulated in blastocysts produced with both concentrations of nobiletin supplementation during MN_EGA_ phase or 10 µM of nobiletin supplementation during MJ_EGA_ compared to controls. These results together are in line with other studies which demonstrated that antioxidant supplementation in IVC medium, like crocetin^23^ and L-carnitine^21^, improved embryo quality by decreasing their lipid content. Regarding nobiletin, studies in mice showed its ability to reduce hepatic lipid accumulation, prevent lipoprotein overproduction and normalize insulin sensitivity when supplied in the diet^32^. Moreover, it has been demonstrated that nobiletin reduces lipid accumulation and regulates lipidic metabolism in hepatic cell lines^17,33^. There is evidence that nobiletin upregulates the expression of *PPARα* in white adipose tissue of mice^17^. An explanation for the reduction of lipids by nobiletin has been proposed indicating that full methoxylation of the A-ring of nobiletin seems to be the most optimal structure to express potent effects on modulating hepatic lipid metabolism via primarily suppressing lipoprotein secretion in HepG2 cells^33^. Therefore, it appears that the ability of nobiletin to reduce lipid content and improve mitochondrial activity in blastocysts may be related to the properties of its chemical structure that allows modulation of lipid metabolism and mitochondrial activity. Moreover, activation of *PPARα* by nobiletin could result in increased embryo lipid turnover through the β-oxidative pathway, preventing accumulation of lipoperoxides despite peroxisomal induction. Recent studies have shown that response of embryos to IVC involves a variety of metabolic factors that act as signals of extracellular and intracellular conditions to which the early embryos can adapt cell programming, signaling pathways, mitochondrial metabolism (mitochondrial production of Acetyl‐Coenzyme A (Acetyl‐Co A) and methyl groups, which are dependent on the availability of glucose, lipids and amino acids) or peroxisome proliferator-activated receptors (PPARs) in response to lipid content. These factors in the embryo are translated into effects on developmental speed or epigenetic modifications^6,34,35^. Consequently, these results can reinforce the antioxidant-defense role of nobiletin during early embryo development in vitro in bovine and could indicate an improvement of the quality of the produced blastocysts.

Embryo cell number is a parameter correlated with embryonic development and quality. Also, it has been reported for different cellular lines (MOLT-4, HUVEC, PC12D, K-N-SH cells) that nobiletin exert its activity by modulation of cell cycle progression^17^. We observed that regardless of EGA phase (MN_EGA_ and MJ_EGA_), nobiletin supplementation in culture media increased the total cell number of produced blastocysts. This increase rate was similar to that observed with other antioxidants such as vitamin C^15^ or crocetin^16,23^, suggesting that nobiletin could directly stimulate the cell cycle during EGA and improve embryo quality.

To verify if the effects of nobiletin during MN_EGA_ or MJ_EGA_ were related to gene expression changes, we analyzed the expression of candidate genes for oxidative stress, embryo development and quality. Glutathione peroxidase (*GPX1*) and Nuclear Factor Erythroid 2-Like 2 (*NFE2L2*), are oxidative-stress-response-related genes. *GPX1*, considered the major antioxidant enzyme within the Glutathione peroxidase family, is ubiquitously expressed in the cytosol and also has been found in mitochondria^20^. Furthermore, *GPX1* acts as a scavenger of hydrophilic peroxide species, can be transformed into an enzymatically inactive cellular structural component, and protects cells against oxidative damage^20^. During in vitro production ROS generation increases and one of the defenses to counter excess ROS in the embryo is *GPX1*; therefore, *GPX1* overexpression has been positively linked with embryo quality^36,37^. In our study, gene expression analysis revealed the upregulation of *GPX1* in 8- and 16-cell embryos as well as in blastocysts produced with nobiletin supplementation during MN_EGA_ or MJ_EGA_ phases. A similar response has been reported in sheep and bovine embryos treated with other types of antioxidants like L-carnitine^21^ or crocetin^16^. *NFE2L2* transcript (also known as *Nrf2*) is important for embryo tolerance to oxidative stress during EGA as well as for its competence for development^2^. PI3K/AKT pathway plays a role in regulating *NFE2L2* activation and is involved in the regulation of protein kinases, which may induce nuclear translocation^38^. Harris and Hansen^39^, and Ghanem et al.^29^ reported in mice that up-regulation of *NFE2L2* transcript may protect embryos from oxidative stress through preservation of intracellular redox states to ensure normal embryonic development. In the same line, our results showed the relative abundance of *NFE2L2* transcript increased in 16-cell stage embryos cultured with 10 µM nobiletin during MJ_EGA_, while remained unaltered in 8-cell stage embryos as well as in blastocysts from both treatments. Moreover, data obtained in cancer cells of mice showed that *NFE2L2* mRNA levels were upregulated when nobiletin was supplemented in culture medium^40^. Taken together, these data suggest that nobiletin plays an antioxidant-defense role via distinct pathways during the different phases of early embryo development in vitro. However, it is necessary to confirm this antioxidant action by measuring ROS levels in the embryos.

Cyclin Dependent Kinase 2 (*CDK2*) is necessary for cell cycle progression, and is a major kinase that governs AKT phosphorylation, while it also participates on EGA^41^. In our study, *CDK2* mRNA expression was upregulated in 8-cell (MN_EGA_), and blastocysts (MJ_EGA_) cultured with 5 µM or 10 µM of nobiletin as well as in 16-cell (MJ_EGA_) cultured with 10 µM of nobiletin. This is in line with previous data obtained in bovine embryos that showed changes in the levels of transcription in genes associated with cell cycle and observed an increase in *CDK2* expression during early embryo development (8 and 16-cell embryos, and blastocysts)^42^. Conversely, studies using nobiletin in cancer cells (U87, Hs683) showed a decrease in *CDK2* expression^17,43^. Hence, nobiletin seems to respond differently depending on the cell type.

Histone H3.3 is encoded by H3.3 histone A (*H3-3A*) and H3.3 histone B (*H3-3B*) genes and is related to DNA synthesis and integrated into embryonic nucleosomes to mark genes for subsequent expression in development^44^. We observed that supplementation of 5 or 10 µM nobiletin during MN_EGA_, increased the expression of *H3-3B* and *H3-3A* genes in 8- cell embryos while supplementation of 10 µM nobiletin during MJ_EGA_ increased the expression of *H3-3B* gene in 16-cell embryos. This is in corroboration with results from a recent study were characterization of the expression of both genes that encode *H3.3* (*H3-3A and H3-3B*) was performed in early bovine embryos, demonstrating that *H3-3B* mRNA is very abundant throughout early embryogenesis, being two to three times higher than *H3-3A* mRNA during the major wave of EGA^45^. Additionally, a higher abundance of *H3-3B* compared to *H3-3A* was found in mouse embryos^46^, suggesting that the protein encoded by *H3-3B* gene may be critical for initiating the transcription of embryonic genes during EGA. As mentioned above, EGA is crucial for further embryo development and regulated by several important factors^47^. One crucial factor is histone modification, including methylation and acetylation^48^.Likewhise, Acetyl‐Co A is a central metabolite linking glucose oxidation and long‐chain fatty acid or cholesterol synthesis, providing energy and materials for cell growth and proliferation. Furthermore, Acetyl‐Co A, as a donor of an acetyl group, can be utilized by histone acetyltransferases for histone acetylation^49^. A recent study showed that Acetyl‐CoA synthases are essential for maintaining histone acetylation under metabolic stress during EGA in pigs and they corroborated that β‐oxidation is crucial for porcine embryo development by contributing to energy metabolism and histone acetylation^50^. This suggests one more time that nobiletin could prefer the β-oxidation pathway as an energy production mechanism.

During in vitro development, embryos have a series of metabolic factors that are required in proliferation, differentiation, and survival of cells^13,51^. In this context, the quality of the embryos produced in vitro depends on many factors, among them the expression of different genes, as we have shown in this study. Gene expression depends of different signaling pathways that play important roles in the formation of the blastocyst, for example, PI3K/AKT^12,13,51^. AKT regulates cellular processes such as glucose metabolism, transcription, cellular growth and proliferation^51^. In blastocysts, AKT inhibition alters their development and AKT activation triggers the differentiation and migration of trophoblast cells^14,52^. Other studies showed that the AKT appear to have an important role in early embryonic development, in double-knockout mice deletion of any of the AKT isoforms leads to embryonic death or exhibiting more severe phenotype and earlier lethality^53^. In addition, PI3K/AKT regulates the development of preimplantation embryo by mediating the effects of autocrine factors^54^. Previous studies in cell lines have shown that nobiletin can act through various signaling pathways, including AKT^17^. However, as far as we know, nobiletin action on AKT pathway in bovine blastocysts produced in vitro is unknown. In this study, we established the presence of the AKT pathway in bovine blastocysts. Based on immunofluorescence images, p-AKT protein appears to be predominantly localized in the cytoplasm of embryos cultured with 5 and 10 µM nobiletin during MN_EGA_ and MJ_EGA_, suggesting constant stimulation of this pathway during the preimplantation period. Expression of the AKT protein and its phosphorylation status were confirmed by western blot analysis of bovine blastocysts produced with or without nobiletin supplementation during MN_EGA_ or MJ_EGA_ phases. Similar results were found by Ashry et al.^14^, who investigated the relationship between AKT signaling and the embryotrophic actions of follistatin, and indicated that it plays an important role in the regulation of AKT signaling in early bovine embryos. Together, these results suggest that nobiletin is associated with increased AKT phosphorylation and, as it has been shown in cell lines studies, nobiletin has the ability to interact with this pathway, and regulate specific genes. In our study increased AKT phosphorylation might be related to the increase in the production and the expression of genes that favor the progression of the cell cycle (*CDK2*) and to the improvement in embryo quality by increasing mitochondrial activity and genes related to oxidative stress (*NFE2L2*), and reducing lipid content (*PPARα*). However, further studies are needed to fully elucidate its mechanism of action in early embryos.

**Figure 8:**
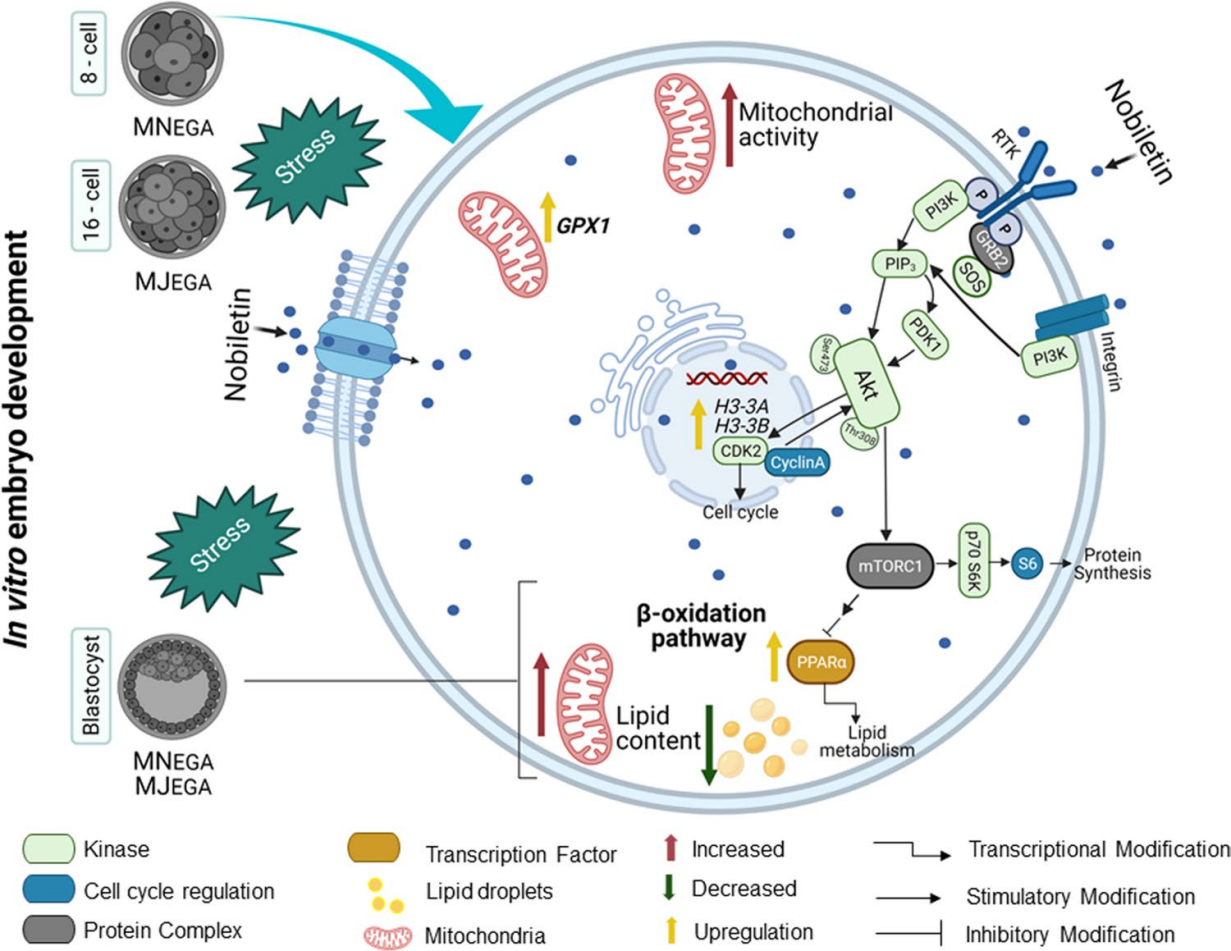
Schematic diagram illustrating the effect of nobiletin on bovine embryo development during the two EGA phases (MN_EGA_ and MJ_EGA_). Nobiletin a class of polymethoxylated flavone, is easily absorbed across the cytoplasmic membranes due to its structure and lipophilic nature^17^, but the specific mechanism of action is not clear yet. Nobiletin supplementation during EGA has a positive effect on preimplantation bovine embryonic development, also increase the abundance of *CDK2* (cell cycle progression), *H3-3A*,*H3-3B* (development) and *GPX1* (oxidative stress) transcripts in 8-cells and 16-cells embryos. In addition, the nobiletin effect in the produced blastocysts was reflected by stimulating mitochondrial activity, decreasing the cytoplasmatic accumulation of lipids and promoting the expression of genes that regulate lipid metabolism and protect against oxidative stress. Besides, these positive responses of nobiletin on embryonic development and quality of the produced blastocysts in vitro could be modulated by activation of the AKT signaling pathway. In our study increased AKT phosphorylation might be related to the increase in the production and the expression of genes that favor the progression of the cell cycle (*CDK2*) and reducing lipid content (*PPARα*). However, further studies are needed to fully elucidate its mechanism of action in early embryos. Figure created with BioRender.com.

In conclusion, nobiletin supplementation during MN_EGA_ or MJ_EGA_ has a positive effect on preimplantation bovine embryonic development in vitro by increasing blastocyst production and also corroborates on the increase in transcription level of genes related to cell division. Besides, this effect is reflected on the blastocysts quality improvement by (i) stimulating mitochondrial activity and expression of genes related to the protection of oxidative stress, and (ii) reducing the cytoplasmatic accumulation of lipids and promoting the expression of genes that regulate lipid metabolism. In addition, these positive responses of nobiletin on embryonic development and quality of the produced blastocysts in vitro could be modulated by the activation of AKT signaling pathway (Fig. 8). Therefore, nobiletin could constitute a suitable supplement to overcome oxidative stress in bovine IVP and improve ARTs in mammals.

## Methods

Unless stated otherwise, all reagents were purchased from Sigma-Aldrich Corporation (St Louis, MO, USA).

### Oocyte collection and maturation

Immature cumulus-oocyte complexes (COCs) were obtained by aspirating follicles (2–8 mm) from the ovaries of mature heifers and cows collected from a local abattoir. COCs (homogeneous cytoplasm and intact CCs) were selected and matured in four-well dishes (Nunc, Roskilde, Denmark) in 500 μL maturation medium (TCM-199), supplemented with 10% (v/v) fetal calf serum (FCS) and 10 ng/mL epidermal growth factor (EGF), in groups of 50 COCs per well for 24 h at 38.5 °C and an atmosphere of 5% CO_2_ in the air with maximum humidity^55^.

### Sperm preparation and in vitro fertilization (IVF)

IVF was performed as described previously^56^. Briefly, frozen semen straws (0.25 mL) from an Asturian Valley bull previously tested for IVF were thawed at 37 °C in a water bath for 1 min and centrifuged for 10 min at 280×g through a gradient of 1 mL of 40% and 1 mL of 80% Bovipure (Nidacon Laboratories AB, Göthenborg, Sweden) according to the manufacturer´s instructions. The sperm pellet was isolated and washed in 3 mL of Boviwash (Nidacon Laboratories AB) by centrifugation at 280×g for 5 min. The pellet was re-suspended in the remaining 300 µL of Boviwash. The final concentration of spermatozoa was adjusted to 1×10^6^ spermatozoa/mL. Gametes were co-incubated for 18–22 h in 500 µL fertilization medium (Tyrode’s medium) with 25 mM bicarbonate, 22 mM sodium lactate, 1 mM sodium pyruvate and 6 mg/mL fatty acid-free bovine serum albumin (BSA) supplemented with 10 mg/mL heparin sodium salt (Calbiochem) in four-well dishes in groups of 50 COCs per well in an atmosphere of 5% CO_2_ in the air with maximum humidity at 38.5 °C.

### In vitro culture of presumptive zygotes

At approximately 21 hpi, a total of 7237 (3398 for MN_EGA_ phase and 3839 for MJ_EGA_ phase) presumptive zygotes were denuded of cumulus cells (CCs) by vortexing for 3 min and then cultured in groups of 50 in a four-well dish containing 500 µL per well of culture medium (synthetic oviductal fluid (SOF);^57^ supplemented with 5% (v/v) FCS, 4.2 mM sodium lactate, 0.73 mM sodium pyruvate, 30 µL/mL basal medium eagles (BME) amino acids, 10 µL/mL minimum essential medium (MEM) amino acids and 1 µg/mL phenol red, in the presence (MN_EGA_) or absence (MJ_EGA_) of nobiletin (MedChemExpress, MCE, Sweden) or with 0.03% dimethyl sulfoxide (DMSO vehicle for nobiletin dilution). At 54 hpi those embryos that reached the 8-cell stage were selected and randomly cultured in groups of 50 in SOF + 5% FCS until Day 8 (MN_EGA_) or in presence of nobiletin or DMSO until 96 hpi (MJ_EGA_). At 96 hpi those embryos that reached the 16-cell stage were selected and randomly cultured in groups of 50 in SOF + 5% FCS until Day 8 (MJ_EGA_) (see ‘Experimental design’ described below for more details). Culture took place at 38.5 °C in an atmosphere of 5% CO_2_, 5% O_2,_ and 90% N_2_.

## Assessment of embryo development and quality

### Embryo development

Developmental rate was recorded at 54 hpi from MN_EGA_ and MJ_EGA_ (≥ 8-cell) and 96 hpi from the MJ_EGA_ phase (≥ 16-cell). For both phases, cumulative blastocyst yields were recorded at Day 7, and 8 pi under a stereomicroscope.

### Embryo quality: mitochondrial activity measurement, lipid content quantification and total cell number of blastocysts

Day 7 blastocysts (~ 30 per group) were simultaneously evaluated regarding mitochondrial activity, the number of lipid droplets, and total cell number. Blastocysts from each treatment were first suspended in 100 µL phosphate-buffered saline (PBS) without calcium or magnesium supplemented with 0.1% polyvinylpyrrolidone (PVP). Next, blastocysts were equilibrated for 15 minutes in culture media supplemented with 5% FCS and then incubated for 30 min at 38.5 °C in 400 nM MitoTracker DeepRed (Molecular Probes, Eugene, USA) for mitochondrial activity; blastocysts were then fixed in 4% paraformaldehyde (PF) for 30 min at room temperature. For lipid content analysis, fixed blastocysts were permeabilized with 0.1% saponin for 30 min and stained for 1 h with 20 μg/mL Bodipy 493/503. For analysis of total cell number, blastocysts were stained with Hoechst 33342 (10 μg/mL) for 30 min, washed in PBS + 0.1% PVP three times for 5 minutes each, and then mounted in 3.8 μL mounting medium between a coverslip and a glass slide which was sealed with nail polish. Slides were examined using a laser-scanning confocal microscope (Leica TCS SP2) equipped with an argon laser excited at 488 nm and with an emission spectrum of 500–537 nm for visualization of lipid droplets. For mitochondria, we used excitation and emission set at 644 nm and 625–665 nm, respectively. All images were captured using the same parameters, performing sequential acquisition.

For the assessment of mitochondrial activity, the fluorescence signal intensity (pixels) was quantified. Serial sections of 5 µm were made for each blastocyst and a maximum projection was accomplished for each one. Images obtained were evaluated using the ImageJ program (NIH; ImageJ version 1.52k software (http://rsbweb.nih.gov/ij/)). After selection using the freehand selection tool, each blastocyst was measured to determine its area and its integrated density (IntDen), which corresponds to pixel intensity. Also, the background fluorescence of an area outside the blastocyst was measured. Fluorescence intensity in each blastocyst was determined using the following formula: Relative fluorescence = IntDen − (area of selected blastocyst x mean fluorescence of background readings). Fluorescence intensities are expressed in arbitrary units (a.u.).

The lipid quantification in blastocysts was obtained by analysis of the total area of lipids in each blastocyst. We captured three images of each blastocyst: one in the middle of the blastocyst (the image with the largest diameter) and the other two in the middle of the resulting halves. We used a 63× objective at a resolution of 1024 × 1024 and images were analyzed using the ‘nucleus counter’ tool, set to detect, distinguish, and quantify droplet areas with the ImageJ program. For blastocysts, lipid quantity was corrected by total embryo area, to account for varying blastocyst sizes. After verification of a significant correlation (r^2^ = 0.84 and *P* < 0.0001 by Pearson’s correlation test) between lipid quantity of three sections in 30 blastocysts (10 per group) we chose the section with the largest area per blastocyst to be analyzed^58^. Simultaneously, the total number of cells per blastocyst was determined by counting the Hoechst stained cells under an epifluorescence microscope (Nikon 141731) equipped with a fluorescent lamp (Nikon HB-10104AF) and UV-1 filter.

### Embryos at 8- and 16-cell stage and blastocysts for gene expression analysis

Gene expression analysis was performed using 3 pools of 10 embryos at 8-cell (MN_EGA_); 16-cell (MJ_EGA_); and Day 7 blastocysts of both phases (MN_EGA_ and MJ_EGA_) per treatment group: Control, C_DMSO_, Nob5, and Nob10. Poly(A) RNA was extracted using the Dynabeads mRNA Direct Extraction Kit (Ambion; Thermo Fisher Scientific) with minor modifications^59^. Immediately after poly(A) RNA extraction, reverse transcription (RT) was performed using a Moloney murine leukemia virus (MMLV) Reverse Transcriptase 1st-Strand cDNA Synthesis Kit according to the manufacturer’s instructions (Epicentre Technologies Corp, Madison, WI, USA). Tubes were heated to 70 °C for 5 min to denature the secondary RNA structure, allowing Poly(T) random primers and Oligo dT annealing, and the RT mix was then completed by adding 0.375 mM dNTPs (Biotools, Madrid, Spain), 6.25 U RNAsin RNAse inhibitor (Promega, Madison, WI, USA), MMLV HP RT 10x reaction buffer, 5 mM DTT and 5 U MMLV high-performance reverse transcriptase (Epicentre Technologies Corp, Madison, WI, USA). Samples were incubated at 25 °C for 10 min, and then at 37 °C for 60 min, to allow the RT of RNA, and finally at 85 °C for 5 min to denature the enzyme. All mRNA transcripts were quantified in duplicate using a Rotorgene 6000 Real-Time Cycler (Corbett Research, Sydney, Australia). RT–quantitative polymerase chain reaction (qPCR) was performed by adding a 2 µL aliquot of each cDNA sample (~ 60 ng µL^−1^) to the PCR mix (GoTaq qPCR Master Mix, Promega) containing specific primers to amplify the genes of interest. Primer sequences are provided in Supplementary Table S4. The selection of genes to be evaluated was carried out considering the expression of key genes in preimplantation embryonic development. All primers were designed using Primer-BLAST software (http://www.ncbi.nlm.nih.gov/tools/primer-blast/) to span exon-exon boundaries when possible. For quantification, RT-qPCR was performed as described previously^60^. The PCR conditions were tested to achieve efficiencies close to 1. Relative expression levels were quantified by the comparative cycle threshold (CT) method^61^. Values were normalized using two housekeeping genes (*H2AFZ* and *ACTB*) selected according to previous studies^56,62^, while their stabilities were evaluated using the geNorm software for microsoft^63,64^, ranking the genes based on the internal control gene stability parameter M. Fluorescence was acquired in each cycle to determine the threshold cycle or the cycle during the log-linear phase of the reaction at which fluorescence increased above background for each sample. Within this region of the amplification curve, a difference of one cycle is equivalent to a doubling of the amplified PCR product. According to the comparative CT method, the ΔCT value was determined by subtracting the mean CT value of the two housekeeping genes from the CT value of the gene of interest in the same sample. The calculation of ΔΔCT involved using the highest treatment ΔCT value (i.e. the treatment with the lowest target expression) as an arbitrary constant to subtract from all other ΔCT sample values. Fold-changes in the relative gene expression of the target were determined using the formula 2^−ΔΔCT.^

### Immunofluorescence of phospho-AKT (p-AKT) in blastocysts

Immunolocalization of p-AKT was performed according to López-Cardona et al.^65^ with minor modifications. Day 7 blastocysts (n = 10 per group) were washed twice with PBS + 0.1% PVP and fixed in 4% PF for 10 min at room temperature. Next, they were permeabilized by incubation in PBS with 10% FCS and 1% Triton X-100 for 45 min at room temperature. After permeabilization, blastocysts were incubated overnight at 4 °C in PBS + 0.1% PVP and 5% FCS and 1:100 rabbit polyclonal antibody against p-AKT (Thr308/Ser473) (D9E) XP® Rabbit mAb (Cell Signaling Technology, #4060). Following incubation, blastocysts were washed twice in PBS + 0.1% PVP and incubated in PBS supplemented with 5% FCS and 1:250 goat anti-rabbit polyclonal antibody Alexa Fluor 488-conjugate (Molecular Probes, Eugene, OR, USA), for 2 h at room temperature followed by washing again three times in PBS + 0.1% PVP. In all cases, nuclei were stained with Hoechst 33342 (10 μg/mL). Finally, blastocysts were mounted in microdrops with Fluoromount G (EMS, Hatfield, UK) and examined by confocal microscopy (Leica TCS-SPE). Negative control was prepared to omit the primary antibody before adding the secondary antibody.

### Western blot of AKT in blastocysts

The western blot analysis was performed as described previously by Ashry et al.^66^ with minor modifications. Day 7- 8 blastocysts (n = 20 blastocysts/group, n = 3 replicates/EGA phase) were lysed in 1 × RIPA buffer (150 mM NaCl, 1% Triton X-100, 0.5% sodium deoxycholate, 0.1% SDS, and 50 mM Tris [pH 7.6]), supplemented with 1 × protease, phosphatase Inhibitor Cocktail (Roche, Basel, Switzerland), for 1 h at 4 °C. The samples were mixed with 1 × sample buffer and then denatured at 95 °C for 10 min. Proteins were resolved by SDS-PAGE (12% acrylamide gel loading 45 µL of total protein per well) and transferred onto a nitrocellulose membrane. After the transfer, membranes were blocked for 30 min in 3% BSA in PBS + 0.1% Tween-20 (PBS-T) at room temperature, and was incubated overnight at 4 °C with a total (t)AKT rabbit polyclonal antibody [1:1000 (Vol:Vol), Cell Signaling Technology, #9272S]; or a p-AKT (Thr308) polyclonal antibody [1:1000 (Vol:Vol), Cell Signaling Technology, #9275S]; or a p-AKT (Ser473) polyclonal antibody [1:1000 (Vol:Vol), Cell Signaling Technology, #9271S]. Then, incubation with the secondary antibody goat anti-rabbit IgG-HRP [1:2500 (Vol:Vol), Cell Signaling Technology, #7074S] was conducted for 2 h at room temperature revealed by Enhanced Chemiluminescence kit (RPN2109, ECL^TM^, Amersham GE Healthcare) and detected by an ImageQuant LAS 500 chemiluminescence CCD camera (GE Healthcare Life Sciences, USA, 29005063). The monoclonal anti-β-actin−peroxidase antibody produced in mouse was used as the loading control.

Membranes were probed sequentially with primary p-AKT (Thr308), p-AKT (Ser473) and (t)AKT antibodies. For this purpose, after detection of an antibody membranes were stripped by washing extensively in TBS-T, three times for 10 minutes each, and repeating the blocking step, and then the membranes are re-probed with the next antibody. After detection of (t)AKT, membranes were stripped and re-probed with anti-β-actin−peroxidase antibody. In all cases, intensities of protein bands (optical density (OD)) were quantified by ImageJ software and the relative abundance of each protein was normalized to the total–actin expression in the corresponding lane and phosphorylation level was expressed as phosphorylated (p) AKT/(t) AKT. The ratio of the OD of the protein concerned (AKT/p-AKT) in relation to actin is presented in the form of bar charts.

### Experimental design

####  Experiment 1: effect of nobiletin on early embryo development in vitro

**Figure 9:**
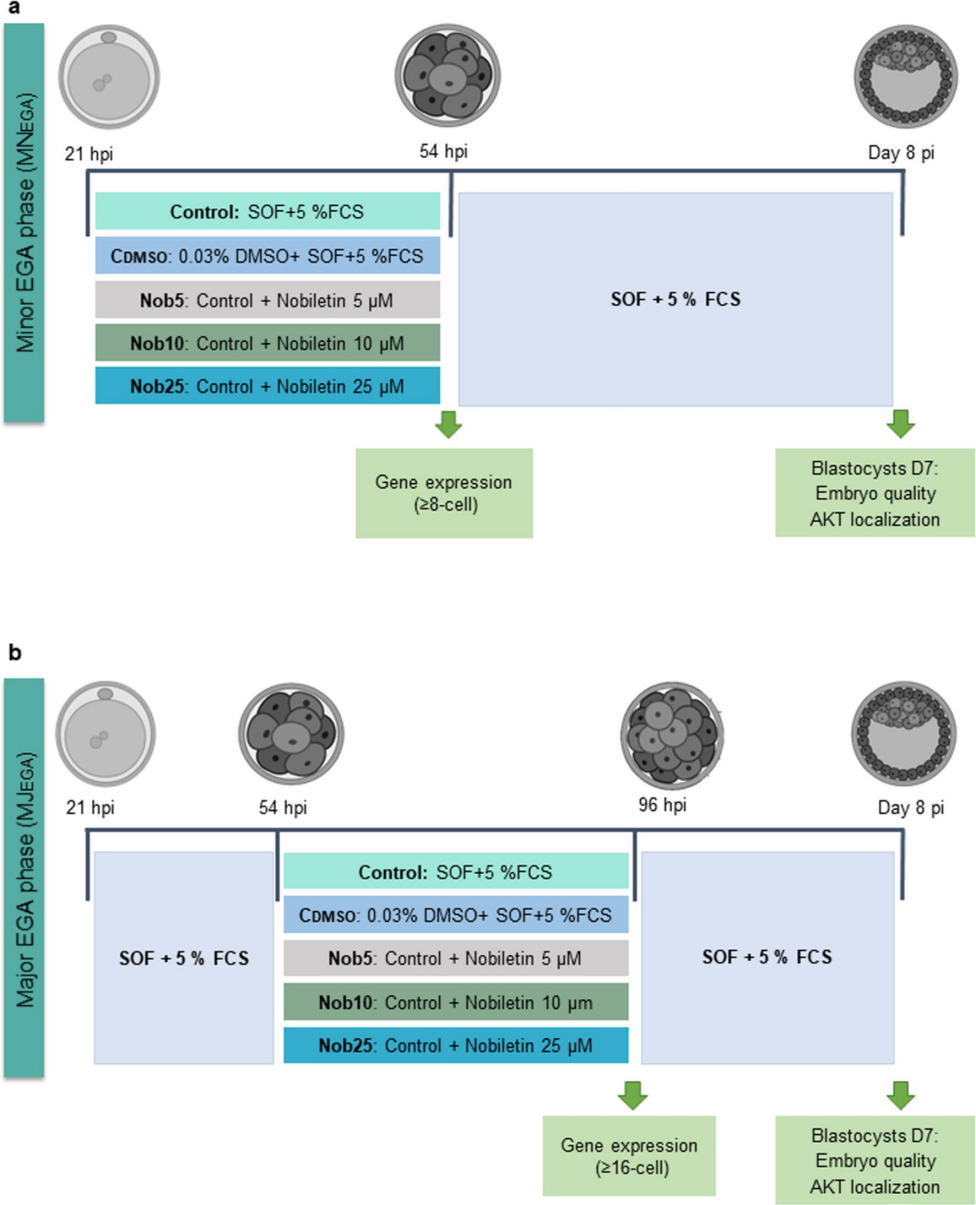
Experimental design. (**a**) MN_EGA_: Presumptive zygotes were cultured during the minor EGA phase (2- to 8-cell stage: 21–54 hpi) in synthetic oviductal fluid (SOF) with 5% fetal calf serum (FCS) (Control); supplemented or not with 5, 10 and 25 µM nobiletin (Nob5, Nob10 and Nob25, respectively), or with 0.03% dimethyl sulfoxide [control for DMSO vehicle for nobiletin dilution (C_DMSO_)]. At 54 hpi, embryos that reached the 8-cell stage were transferred to SOF + 5% FCS and cultured until Day 8 maintaining each experimental group separately. (**b**) MJ_EGA_: Presumptive zygotes were cultured in SOF + 5% FCS (Control) until 54 hpi. At 54 hpi, embryos that reached the 8-cell stage were cultured during the major EGA phase (8-cell to 16-cell stage: 54–96 hpi) in SOF + 5% FCS supplemented or not with Nob5, Nob10 and Nob25, or with 0.03% dimethyl sulfoxide (C_DMSO_). At 96 hpi, embryos that reached the 16-cell stage were transferred to SOF + 5% FCS and cultured until Day 8 maintaining each experimental group separately.

In this experiment the effect of nobiletin supplementation on embryo development during two developmental periods: (a) MN_EGA_: from 2-cell to 8-cell stage, minor EGA phase; and (b) MJ_EGA_: from 8-cell to 16-cell stage, major EGA phase was determined by evaluating the cleavage rate at 54 hpi and blastocysts yield at Days 7 and 8 (Fig. 9).

For this purpose, presumptive zygotes/embryos from 2- to 8- cell stage (MN_EGA_: 21-54 hpi) or embryos from 8- to 16-cell stage (MJ_EGA_: 54–96 hpi) were cultured in SOF + 5% FCS alone (Control: n = 730 and 621 for MN_EGA_ and MJ_EGA_ respectively) or supplemented with 5, 10 or 25 µM nobiletin (Nob5: n = 757 and 518; Nob10: n = 695 and 553; and Nob25: n = 521 and 424 for MN_EGA_ and MJ_EGA_ respectively), or 0.03% DMSO (C_DMSO_: n = 695 and 622 for MN_EGA_ and MJ_EGA_ respectively). For MJ_EGA_ phase groups, embryo culture until 8-cell stage (21-54 hpi) was performed in SOF + 5% FCS. At 54 hpi (MN_EGA_ - Control: n = 388; Nob5: n = 386; Nob10: n = 352; Nob25: n = 254; C_DMSO_: n = 368) or 96 hpi (MJ_EGA_- Control: n = 331; Nob5: n = 315; Nob10: n = 347; Nob25: n = 210; C_DMSO_: n = 331), embryos that reached the 8- or 16- cell stage, respectively, were transferred to SOF + 5% FCS and cultured until Day 8, maintaining the different experimental groups separately (Fig. 9). Embryos were cultured in groups of 50 under an atmosphere of 5% CO_2_, 5% O_2_ and 90% N_2_ at 38.5 °C.

Considering that during the experiment it was necessary to preselect the embryos at different stages of development (≥ 8 cells and ≥ 16 cells), the developmental parameters were calculated as follows: (I) developmental rate at 54 hpi: percentage of presumptive zygotes that developed to the 8-cell stage; (II) developmental rate at 96 hpi: percentage of selected 8-cell embryos at 54 hpi that developed to the 16-cell stage; and (III) blastocyst yield: percentage of selected 8-cell embryos (54 hpi) or 16-cell embryos (96 hpi) that continued in culture and developed to the blastocyst stage at Day 7 and 8. A representative number of 8-cell (MN_EGA_), 16-cell (MJ_EGA_), and Day 7 blastocysts from both phases for each experimental group were frozen in liquid nitrogen (LN_2_) in three groups of 10 and stored at − 80 °C for gene expression (See Experiment 2). Additionally, Day 7 blastocysts from both phases were selected to (i) evaluate quality (See Experiment 2); (ii) immunolocalization of p-AKT or (iii) for western blot analysis (See Experiment 3).

Twelve and ten replicates for MN_EGA_ and MJ_EGA_ phases, respectively, were performed under the same assay conditions.

Only the experimental groups that showed higher blastocyst yield in this experiment (Nob5 and Nob10) in comparison with both control groups (Control and C_DMSO_) were used for experiments 2 and 3.

#### Experiment 2: effect of nobiletin on the quality of in vitro produced blastocysts

To evaluate blastocyst quality of embryos produced in vitro with or without nobiletin supplementation during MN_EGA_ or MJ_EGA_, a representative number of Day 7 blastocysts (n ≈ 30 per group/Experiment 1) were stained with MitoTracker DeepRed, Bodipy and Hoescht to evaluate mitochondrial activity (intensity recorded in arbitrary units (a.u)), lipid content (lipid droplet area in μm^2^) and total cell numbers, respectively. Blastocysts were examined using a laser-scanning confocal microscope or an epifluorescence microscope and images obtained were evaluated using the ImageJ program.

To evaluate if nobiletin induces changes in the expression levels of genes related to embryo development and quality, three independent pools of 10 embryos per stage (8-cell, 16-cell, and blastocyst) obtained from each experimental group cultured with or without nobiletin during MN_EGA_ or MJ_EGA_ (Experiment 1), were used for gene expression analysis by qRT-PCR according to the procedures described above.

The selected genes have been linked to embryonic development and are essential in cell proliferation, differentiation, and embryo quality, such as *PPARG* coactivator 1 alpha (*PPARGC1A*); Peroxisome Proliferator-Activated Receptor Alpha (*PPARα*); Ribosomal Protein S6 Kinase Beta-1 (*RPS6KB1*); Cyclin Dependent Kinase 2 (*CDK2*); H3 Histone Family Member 3B (*H3-3B*) and H3 Histone Family Member 3A (*H3-3A*), including Nuclear Factor Erythroid 2-Like 2 (*NFE2L2*) and Glutathione Peroxidase 1 (*GPX1)* related with oxidative stress.

#### Experiment 3: nobiletin effect on the AKT pathway in blastocysts produced in vitro

To assess if nobiletin can interact with AKT pathway during in vitro embryo development, Day 7 blastocysts (n = 10 - Experiment 1) from each group were stained with p-AKT (Thr308/Ser473) for immunolocalization. To evaluate the phosphorylation level of AKT (Thr308 and Ser473), Day 7 blastocyst (n = 60 - Experiment 1) from each group were frozen in LN_2_ for western blot analysis.

### Statistical analysis

All statistical tests were performed using the software package SigmaStat (Systat Software Inc., San Jose, CA, USA). Cleavage rate, blastocyst yield, mitochondrial activity, lipid content, number of cells per blastocyst, relative mRNA abundance levels, and AKT phosphorylation level, were normally distributed with homogeneous variance, so one-way analysis of variance (ANOVA) with arcsine data transformation, followed by Tukey´s test, was performed to evaluate the significance of differences between groups. The correlation analysis for lipid quantification in blastocysts was determined by Pearson’s correlation coefficient test. Values were considered significantly different at *P* < 0.05. Unless otherwise indicated, data are presented as the mean ± s.e.m.

## Supplementary Information

Supplementary Information.
