# Supplementary material for: Nobiletin enhances the development and quality of bovine embryos in vitro during two key periods of embryonic genome activation
Source: Sci Rep. 2021 Jun 3;11:11796. doi: 10.1038/s41598-021-91158-7 (PMC8175487; doi:10.1038/s41598-021-91158-7)
Supplement: Supplementary file 1 — Supplementary Information. [file 41598_2021_91158_MOESM1_ESM.docx]

**Supplementary Information**

**Nobiletin enhances the development and quality of bovine embryos *in vitro* during two key periods of embryonic genome activation**

# Karina Cañón-Beltrán^1,2§^, Yulia N Cajas^1§^, Serafín Peréz-Cerezales^1^, Claudia LV Leal^1,3^, [Ekaitz Agirregoit](https://onlinelibrary.wiley.com/action/doSearch?ContribAuthorStored=Agirregoitia%2C+Ekaitz)ia^4^, Alfonso Gutierrez-Adán^1^, Encina M González^5^ and Dimitrios Rizos^1^*

1Department of Animal Reproduction, National Institute for Agriculture and Food Research and Technology (INIA), Madrid, 28040, Spain.

2Departamento de Ciencias Biológicas, Universidad Técnica Particular de Loja, Loja, 110107, Ecuador

3Department of Veterinary Medicine, Faculty of Animal Science and Food Engineering, University of São Paulo, Pirassununga, 13635-900, Brazil.

4Department of Physiology, Faculty of Medicine and Nursing, Universidad del País Vasco / Euskal Herriko Unibertsitatea (UPV/EHU), Bizkaia, 48940, Spain

5Department of Anatomy and Embryology, Veterinary Faculty, Complutense University of Madrid (UCM), Madrid, 28040, Spain

*Email address: drizos@inia.es

§These authors contributed equally to this work

Supplementary Table 1. Cleavage rate and kinetics of development at 54 h post-insemination and cumulative blastocyst rates on Days 7 and 8 after *in vitro* culture with or without nobiletin supplemented during MNEGA.

|  | **IVC**  **N** | **Total cleaved**  **54 hpi**  **N**  **(%±s.e.m)** | **< 8 cells**  **N**  **(%±s.e.m.)** | **≥ 8 cells**  **N**  **(%±s.e.m.)** | **IVC**  **54 hpi**  **N** | **Blastocysts** | |
| --- | --- | --- | --- | --- | --- | --- | --- |
|  |  |  |  |  |  | **D7** | **D8** |
|  |  |  |  |  |  | **N**  **(%±s.e.m)** | **N**  **(%±s.e.m)** |
| **Control** | 730 | 602  (82.3±1.0) | 164  (22.6±0.9) | 438  (59.7±1.1) | 388 | 126  (32.7±0.7)^b^ | 132  (34.6±0.7)^b^ |
| **CDMSO** | 695 | 595  (85.5±0.5) | 177  (25.1±0.8) | 418  (60.4±0.7) | 368 | 120  (32.8±0.5)^b^ | 128  (34.9±0.4)^b^ |
| **Nob5** | 757 | 625  (82.4±1.0) | 189  (25.3±0.8) | 436  (57.1±1.4) | 386 | 151  (39.7±0.8)^a^ | 163  (42.7±1.0)^a^ |
| **Nob10** | 695 | 586  (84.5±0.6) | 184  (26.3±0.8) | 402  (58.2±1.1) | 352 | 144  (41.0±1.0)^a^ | 157  (44.4±1.3)^a^ |
| **Nob25** | 521 | 442  (85.0±1.0) | 138  (26.6±1.2) | 304  (58.5±1.6) | 254 | 81  (31.8±1.7)^b^ | 89  (34.6±1.2)^b^ |

MNEGA: embryos cultured from zygotes to 8-cell stage (21–54 hpi) in SOF+5% FCS (Control), supplemented or not with 5, 10 and 25 µM of nobiletin (Nob5, Nob10 and Nob25, respectively), or with 0.03% dimethyl sulfoxide (CDMSO). IVC - N: number of presumptive zygotes in culture. IVC - 54 hpi - N: number of 8-cell embryos in culture at 54 hpi. Blastocysts yield on D7 and D8 is calculated from the 8-cell embryos in culture at 54 hpi. Data are the mean ± s.e.m. Within columns, different superscript letters indicate significant difference (P<0.001) between treatments.

Supplementary Table 2. Cleavage rate and kinetics of development at 54 and 96 h post-insemination and cumulative blastocyst rates on Days 7 and 8 after *in vitro* culture with or without nobiletin supplemented during MJEGA.

|  | **IVC**  **N** | **Total cleaved**  **54 hpi**  **N**  **(%±s.e.m.)** | **< 8 cells**  **N**  **(%±s.e.m.)** | **≥ 8 cells**  **N**  **(%±s.e.m.)** | **IVC**  **54 hpi**  **N** | **< 16 cells**  **N**  **(%±s.e.m.)** | **≥ 16 cells**  **N**  **(%±s.e.m.)** | **IVC**  **96 hpi**  **N** | **Blastocysts** | |
| --- | --- | --- | --- | --- | --- | --- | --- | --- | --- | --- |
|  |  |  |  |  |  |  |  |  | **D7** | **D8** |
|  |  |  |  |  |  |  |  |  | **N**  **(%±s.e.m.)** | **N**  **(%±s.e.m.)** |
| **Control** | 867 | 754  (87.0±0.5) | 133  (15.6±0.3) | 621  (71.4±0.7) | 621 | 250  (40.0±0.4)^a^ | 371  (60.0±0.4)^b^ | 331 | 126  (38.4±1.1)^c^ | 154 (47.3±1.4)^c^ |
| **CDMSO** | 878 | 761  (86.8±0.7) | 139  (16.3±0.5) | 622  (70.5±0.7) | 622 | 246  (39.3±0.4)^a^ | 376  (60.7±0.4)^b^ | 331 | 120  (35.8±1.0)^c^ | 146 (44.0±1.1)^c^ |
| **Nob5** | 724 | 623  (86.4±0.5) | 105  (14.9±0.9) | 518  (71.5±0.9) | 518 | 153  (29.9±0.5)^b^ | 365  (70.1±0.5)^a^ | 315 | 148  (46.6±0.8)^b^ | 169 (52.5±1.5)^b^ |
| **Nob10** | 773 | 667  (86.3±0.4) | 114  (14.8±0.6) | 553  (71.5±0.7) | 553 | 166  (30.1±0.4)^b^ | 387  (69.9±0.4)^a^ | 347 | 190  (54.5±1.1)^a^ | 210 (61.0±0.8)^a^ |
| **Nob25** | 597 | 516  (86.2±0.6) | 92  (15.7±0.9) | 424  (70.5±1.3) | 424 | 166  (39.2±0.8)^a^ | 259  (60.8±0.8)^b^ | 210 | 74  (35.9±1.5)^c^ | 88 (42.5±1.3)^c^ |

MJEGA: embryos cultured from 8-cell to 16-cell stage (54–96 hpi) in SOF+5% FCS (Control), supplemented or not with 5, 10 and 25 µM of nobiletin (Nob5, Nob10 and Nob25, respectively), or with 0.03% dimethyl sulfoxide (CDMSO). IVC - N: number of presumptive zygotes in culture. IVC - 54 hpi - N: number of 8-cell embryos in culture at 54 hpi. IVC - 96 hpi - N: number of 16-cell embryos in culture at 96 hpi. Blastocysts yield on D7 and D8 is calculated from the 16-cell embryos in culture at 96 hpi. Data are the mean ± s.e.m. Within columns, different superscript letters indicate significant difference (P<0.001) between treatments.

Supplementary Table 3**.** Total nuclei number in *in vitro* produced Day 7 bovine blastocysts produced with or without nobiletin supplemented during MNEGA or MJEGA.

|  | **MNEGA** | | **MJEGA** | |  |
| --- | --- | --- | --- | --- | --- |
|  | No. of embryos examined | Total no. of cells/blastocyst | No. of embryos examined | Total no. of cells/blastocyst | |
| **Control** | 27 | 105.7±0.7^c^ | 33 | 104.9±0.7^b^ | |
| **CDMSO** | 26 | 106.4±0.8^c^ | 30 | 104.6±0.6^b^ | |
| **Nob5** | 25 | 137.3±0.6^a^ | 36 | 133.2±0.9^a^ | |
| **Nob10** | 29 | 126.7±0.8^b^ | 34 | 134.2±0.7^a^ | |

MNEGA: embryos cultured from zygotes to 8-cell stage (21–54 hpi) and MJEGA: embryos cultured from 8-cell to 16-cell stage (54–96 hpi) in SOF+5% FCS (Control), supplemented or not with 5 (Nob5) or 10 µM (Nob10) of nobiletin or with 0.03% dimethyl sulfoxide (CDMSO). Data are the mean ± s.e.m. Within columns, different superscript letters indicate significant difference (P<0.05) between treatments.

Supplementary Table 4. Details of primers used for qRT-PCR analysis

| **Gene symbol** | **Gene name** | **Primer sequence (5’- 3’)** | **Fragment size (bp)** | **GenBank accession number** |
| --- | --- | --- | --- | --- |
| *ACTB* | Actin, beta | F-GAGAAGCTCTGCTACGTCG  R-CCAGACAGCACCGTGTTGG | 264 | AF191490.1 |
| *CDK2* | Cyclin Dependent Kinase 2 | TCTTTGCTGAGATGGTGACCC  GTTAGGGTCGTAGTGCAGCAT | 242 | NM_001014934.1 |
| *GPX1* | Glutathione Peroxidase 1 | F-GCAACCAGTTTGGGCATCA  R-CTCGCACTTTTCGAAGAGCATA | 116 | NM_174076.3 |
| *H2AFZ* | H2A histone family, member Z | F- AGGACGACTAGCCATGGACGTGTG  R- CCACCACCAGCAATTGTAGCCTTG | 209 | NM_174809 |
| *H3-3A* | H3 Histone Family Member 3A | TTTTTCCATGGGGTCAAAAG  TGGAAAAACTGCCAATACCTG | 96 | NM_001014389.2 |
| *H3-3B* | H3 Histone Family Member 3B | GTGGTGGGGAGTGTTGTCTT  AACGCGCAAAGCATTTTACT | 99 | NM_001242571.2 |
| *NFE2L2* | Nuclear Factor Erythroid 2-Like 2 | CAGGACATTGAGCAAGTTTGG  GTGGAAAGGATGCTGTTGAAG | 234 | NM_001011678.2 |
| *PPARGC1A* | PPARG coactivator 1 alpha | AAAAGCCACAAAGACGTCCG  TCTGCTGCTGTTCCGGTTCT | 111 | NM_177945 |
| *PPARα* | Peroxisome Proliferator-Activated Receptor Alpha | CAACCCGCCTTTCGTCATCCAC  TCCGCCTCCTTGTTCTGGATGC | 102 | NM_001034036.1 |
| *RPS6KB1* | Ribosomal Protein S6 Kinase Beta-1 | GGGATAGAGCAGATGGACGTG  TGGTCGTTTGGAGATCATGGG | 117 | NM_205816.1 |


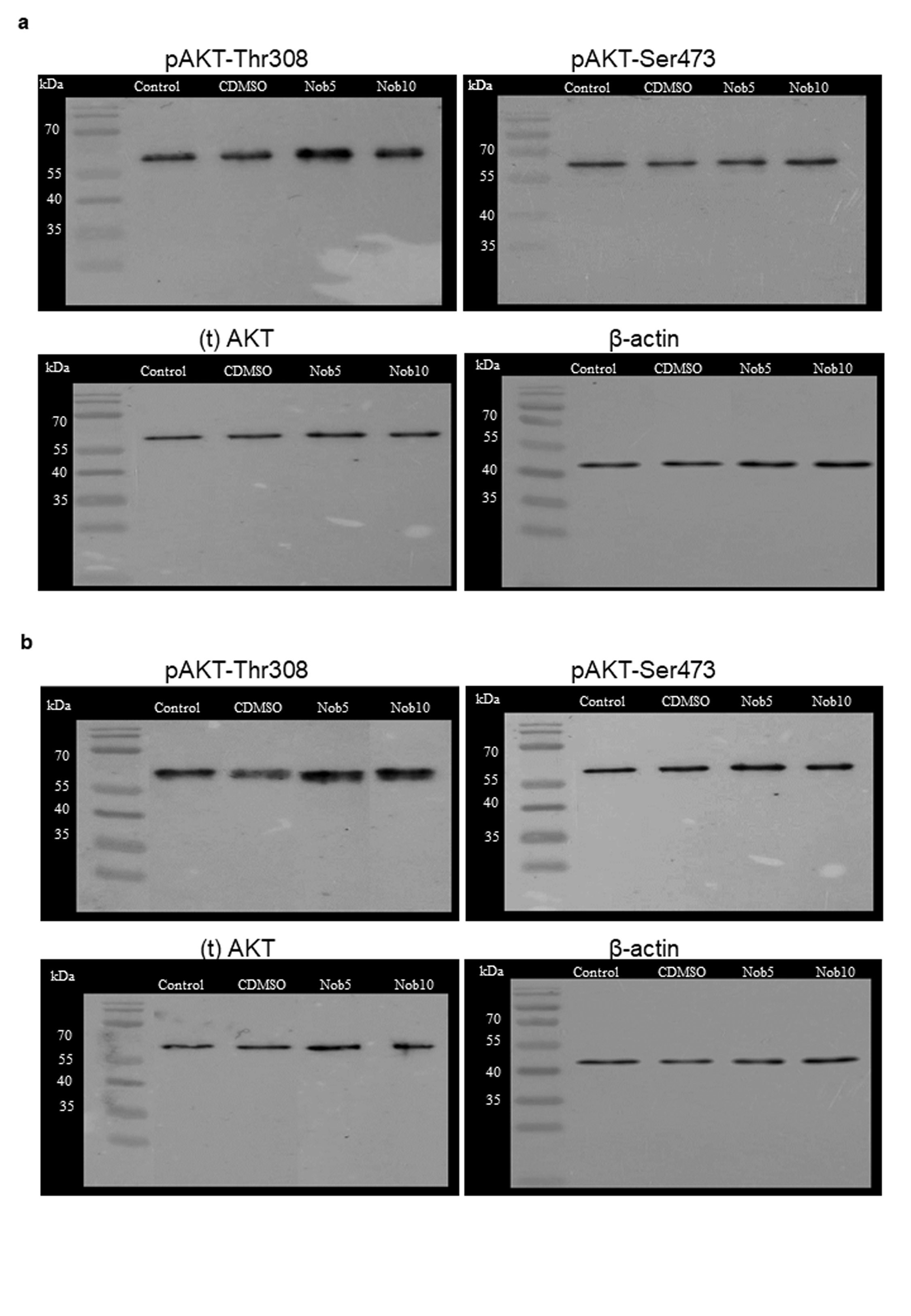


Supplementary Figure 1. Nobiletin increases AKT phosphorylation in *in vitro* produced bovine blastocysts: (a) cultured during 21 - 54 h post-insemination (MNEGA: presumptive zygote to 8-cell stage) or (b) during 54 - 96 hpi (MJEGA: 8- to 16-cell stage) in SOF+5% FCS (Control), with or without 5 µM (Nob5) or 10 µM (Nob10) of nobiletin or with 0.03% dimethyl sulfoxide (CDMSO). Original, uncropped western blot gel images of pAKT-Thr308, pAKT-Ser473, (t)AKT linked to Figure 7. β-actin served as loading control. The Page Ruler Prestained Protein ladder (10–180 kDa) was used as protein size marker.
